# Metabolomics Reveals that Cysteine Metabolism Plays a Role in Celastrol-Induced Mitochondrial Apoptosis in HL-60 and NB-4 Cells

**DOI:** 10.1038/s41598-019-57312-y

**Published:** 2020-01-16

**Authors:** Minjian Chen, Jing Yang, Lei Li, Yanhui Hu, Xiaomei Lu, Rongli Sun, Yubang Wang, Xinru Wang, Xiaoling Zhang

**Affiliations:** 1 State Key Laboratory of Reproductive Medicine, Center for Global Health, School of Public Health, Nanjing Medical University, Nanjing, 211166 China; 2 Key Laboratory of Modern Toxicology of Ministry of Education, Nanjing Medical University, Nanjing, 211166 China; 3 Wuxi Maternal and Child Health Hospital Affiliated to Nanjing Medical University, Wuxi, 214002 China; 4 Experiment Center for Teaching and Learning, Shanghai University of Traditional Chinese Medicine, Shanghai, 201203 China; 5 Department of Hygienic Analysis and Detection, Nanjing Medical University, Nanjing, 211166 China; 6 Safety Assessment and Research Center for Drug, Pesticide, and Veterinary Drug of Jiangsu Province, School of Public Health, Nanjing Medical University, Nanjing, 211166 China; 7 Key Laboratory of Environmental Medicine Engineering, Ministry of Education, School of Public Health, Southeast University, Nanjing, 210009 China

**Keywords:** Cancer metabolism, Molecular biology

## Abstract

Recently, celastrol has shown great potential for inducing apoptosis in acute myeloid leukemia cells, especially acute promyelocytic leukaemia cells. However, the mechanism is poorly understood. Metabolomics provides an overall understanding of metabolic mechanisms to illustrate celastrol's mechanism of action. We treated both nude mice bearing HL-60 cell xenografts *in vivo* and HL-60 cells as well as NB-4 cells *in vitro* with celastrol. Ultra-performance liquid chromatography coupled with mass spectrometry was used for metabolomics analysis of HL-60 cells *in vivo* and for targeted L-cysteine analysis in HL-60 and NB-4 cells *in vitro*. Flow cytometric analysis was performed to assess mitochondrial membrane potential, reactive oxygen species and apoptosis. Western blotting was conducted to detect the p53, Bax, cleaved caspase 9 and cleaved caspase 3 proteins. Celastrol inhibited tumour growth, induced apoptosis, and upregulated pro-apoptotic proteins in the xenograft tumour mouse model. Metabolomics showed that cysteine metabolism was the key metabolic alteration after celastrol treatment in HL-60 cells *in vivo*. Celastrol decreased L-cysteine in HL-60 cells. Acetylcysteine supplementation reversed reactive oxygen species accumulation and apoptosis induced by celastrol and reversed the dramatic decrease in the mitochondrial membrane potential and upregulation of pro-apoptotic proteins in HL-60 cells. In NB-4 cells, celastrol decreased L-cysteine, and acetylcysteine reversed celastrol-induced reactive oxygen species accumulation and apoptosis. We are the first to identify the involvement of a cysteine metabolism/reactive oxygen species/p53/Bax/caspase 9/caspase 3 pathway in celastrol-triggered mitochondrial apoptosis in HL-60 and NB-4 cells, providing a novel underlying mechanism through which celastrol could be used to treat acute myeloid leukaemia, especially acute promyelocytic leukaemia.

## Introduction

Acute leukemia is the most common malignant hematological malignancy, of which acute myeloid leukemia (AML) accounts for over 80% of all acute leukemias in individuals aged >18 years^1^. Chemotherapy is the main treatment of AML. There are still problems such as drug resistance and relapse of leukemia after drug withdrawal, which seriously affect the quality of life and long-term survival of patients. Therefore, it is of great significance to find effective new antineoplastic drugs and explore new therapeutic schemes.

Acute promyelocytic leukaemia (APL), a unique subtype of acute myeloid leukaemia (AML), is a devastating disease characterized by neoplastic proliferation of cells in the bone marrow that have a promyelocytic phenotype and fail to terminally differentiate^2^. Unlike other forms of AML, APL can cause diffuse intravascular coagulopathy and early haemorrhagic death if not promptly diagnosed^3,4^. With the introduction of all-trans retinoic acid (ATRA) and arsenic trioxide (ATO), APL treatment has achieved good outcomes. However, ATRA and ATO can lead to the development of differentiation syndrome and multiorgan toxicity, and a few patients do not respond favourably to the drugs^5,6^. Thus, the, development of less toxic and more effective agents to treat APL is urgent^7^

Traditional Chinese medicine, as an important source of therapeutically effective drugs, has attracted increasing attention in cancer therapy^8^. Recently, celastrol an active component of *Tripterygium wilfordii* Hook f. (Supplementary Material: Fig. S1), is receiving increasing attention. Celastrol has shown great potential for the treatment of inflammation^9^, Gaucher disease^8^, obesity^10^ and multiple cancers, such as prostate cancer^11^, breast cancer^12^, and leukaemia^13^.

The investigation of the effect of celastrol on AML and APL is an interesting topic, and has been widely conducted. Celastrol could significantly prolong the survival of mice in HoxA9/Meis1-induced AML model and APL model^13,14^. In fresh cells from patients of various types of AML, celastrol showed effect for the treatment of leukemia^13,15^. In addition, celastrol could eradicate leukemia stem cell which is the key cause of relapse^16,17^. Importantly, the previous study has demonstrated that celastrol showed stronger anti-tumour effect than ATRA in leukemia cells^13^. Celastrol is also found as a promising and unique agent for managing the sid e effects of ATRA application on APL^18^. Interestingly, the anti-tumour effects of celastrol have been consistently attributed to its ability to induce apoptosis in AML and APL NB-4 cells^15,19–21^, but the mechanism is poorly understood.

HL-60 cells is a widely used model system for studying the molecular events of AML, which lack the t(15;17) translocation characteristic of most cases of APL^13,22,23^. However, HL-60 can respond to ATRA^22^, which is widely used as a cell line in the APL studies^24–27^. In our previous study^28^, consistent to previous reports^15,19–21^, we also found celastrol caused apoptosis in HL-60 cells, indicating the key role of apoptosis in the effect of celastol in the treatment of acute leukemia.

Metabolomics, the systematic measurement and biological interpretation of metabolites within a biological sample, is used to study small molecules and is an integral technology for understanding the function of biological systems. Surveying these small molecules provides an overall understanding of biological mechanisms, thereby creating a more complete picture of the phenotype (the observable characteristics of a living system). In our previous study, we used metabolomics to study the underlying mechanism in HL-60 cells *in vitro*, and identified uridine was the most notable changed metabolite after celastrol treatment^28^. However, the metabolome changes *in vivo* are unknown. As we know, pathogenesis and therapeutic target of leukemia may be not limited in one pathway. Different and complementary conclusions may be reached by using omics analyses of *in vitro* and *in vivo* samples. The hypothesis of this study was that key metabolism changes extracted from metabolome of animal model could reveal mechanism underlying celastrol-induced apoptosis in AML, especially APL. Therefore, in the present study, we treated xenograft HL-60 cell-bearing nude mice with celastrol and used metabolomics to identify the key metabolic changes in tumour tissues *in vivo*. To validate the key findings with the focus on APL, we also applied NB-4 cell according to previous report^26^. NB-4 is a APL cell line, which has the t(15;17) translocation and also has response to ATRA^26^^.^ The role of key metabolic pathway in apoptosis was verified by disrupting the key metabolic changes in both cell lines, revealing that cysteine metabolism plays a key role in celastrol-induced ROS-dependent mitochondrial apoptosis in HL-60 and NB-4 cells.

## Results

### Celastrol inhibited tumour growth and increased apoptotic gene expression in HL-60 cells *in vivo*

**Figure 1:**
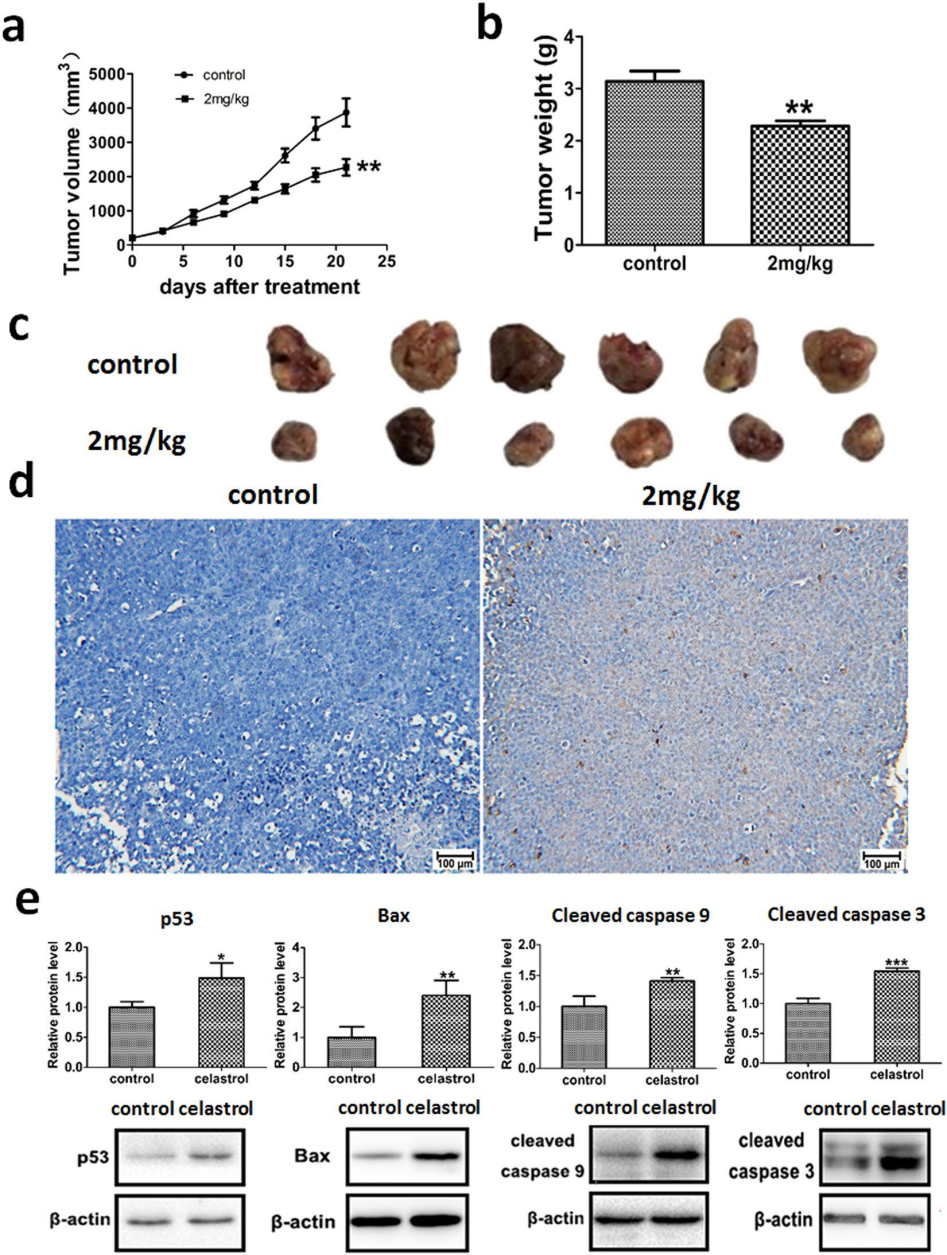
The effects of celastrol on tumour growth and apoptosis *in vivo*. (**a**) The effect of celastrol on tumour volume growth. (**b**) The effect of celastrol on tumour weight. (**c**) The representative graph of the effect of celastrol on tumour volume growth. **(d**) TUNEL assay detecting the effect of celastrol on tumour cell apoptosis. (**e**) The effect of celastrol on the protein levels of apoptosis-related genes in HL-60 cells *in vivo*. *p < 0.05 when compared with control.

In the nude mouse xenograft tumour model, 2 mg/kg celastrol decreased tumour volume and weight (Fig. 1a–c). The TUNEL assay results showed that celastrol induced apoptosis in tumours (Fig. 1d). In addition, we found increased protein levels of p53, Bax, cleaved caspase 9 and cleaved caspase 3 in the celastrol treatment group *in vivo* (Figs. 1e, S2–S5).

### Metabolomics profiles showed that cysteine metabolism was the key metabolic alteration after celastrol treatment in HL-60 cells *in vivo*

**Figure 2:**
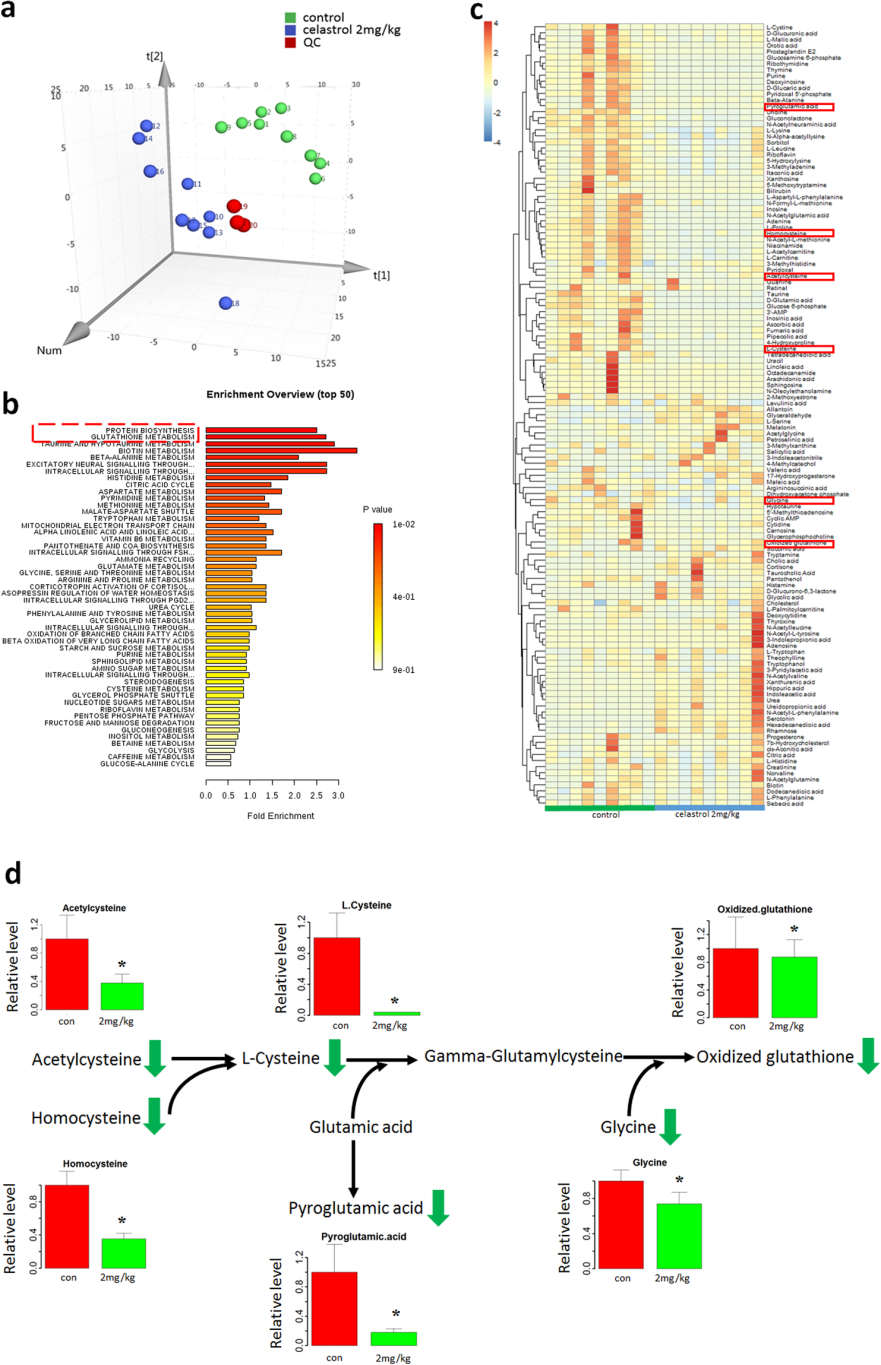
Cysteine metabolism was the key changed metabolism after celastrol treatment. (**a**) 3D PCA model showed good separation between control and celastrol-treated group *in vivo*. (**b**) Pathway enrichment analysis of the metabolomics data *in vivo*. The red indicated significant enriched pathways. (**c**) Heatmap of the metabolomics data in vivo. The metabolites in the red boxes were related to cysteine metabolism. (**d**) Cysteine metabolism alteration after celastrol treatment in HL-60 cells in vivo. Green arrows indicates decrease in vivo. The bar chart shows the statistical results from in vivo study. *p < 0.05 when compared with control.

A total of 129 metabolites were found in tumour tissues (Supplementary Material: Fig. S6). After false discovery rate (FDR) correction, 121 metabolites were found to be significantly changed (q<0.05) (Supplementary Material: Table S1). A total of 43 metabolites significantly increased, while 78 significantly decreased. Principal component analysis (PCA) is an unbiased and unsupervised model that can reveal global celastrol-induced metabolic changes in tumour tissue. We established a 3D PCA model and found good separation between the treatment and control groups (Fig. 2a). The above results indicate that along with the significant growth-inhibitory and pro-apoptotic effect of celastrol on tumour tissue, the metabolome was also dramatically disrupted in tumour tissue, a quite important finding in the search for the underlying mechanism. Among the metabolites, L-cysteine and adenine showed the most marked change. These metabolites were significantly decreased—indeed, totally undetectable—after celastrol treatment in tumour tissues, indicating the importance of L-cysteine metabolism and adenine metabolism in celastrol-induced apoptosis in tumour tissues. To further identify key metabolic changes, the changed metabolites were imported into the “enrichment analysis” module (www.metaboanalyst.ca/), and protein biosynthesis and glutathione metabolism were identified as the key changed pathways (Fig. 2b, Supplementary Material: Table S2). Importantly, glutathione metabolism was directly linked to L-cysteine metabolism; in addition, acetylcysteine and homocysteine, the upstream metabolites of L-cysteine metabolism, were also significantly decreased after celastrol treatment (Fig. 2c,d). These results indicated the importance of cysteine metabolism in HL-60 cells *in vivo* after celastrol treatment. Raw metabolomic data can be found in the Supplementary Material: Table S3.

### Celastrol decreased L-cysteine levels and induced significant accumulation of ROS in HL-60 cells, which could be reversed by acetylcysteine

**Figure 3:**
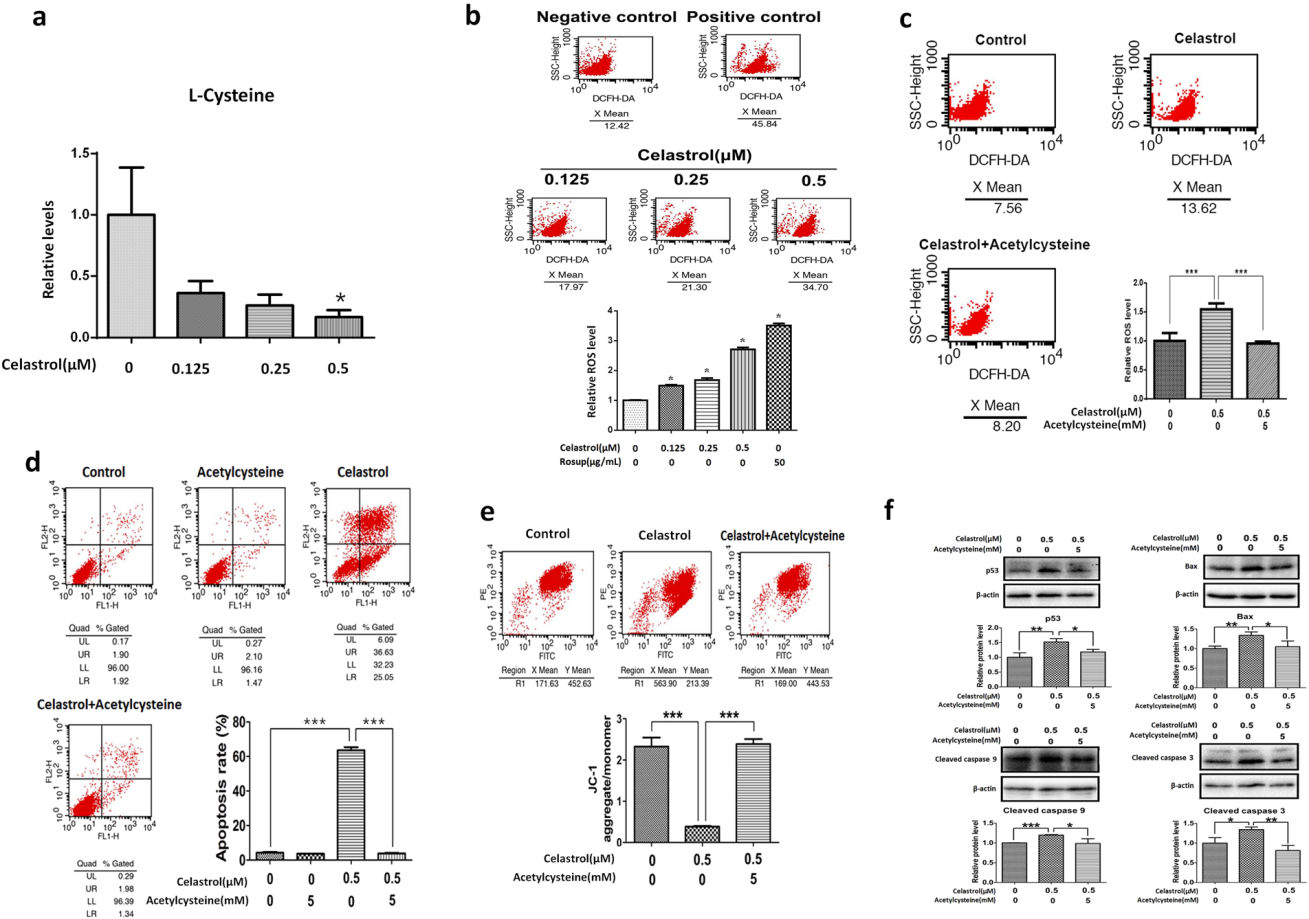
The effects of celastrol on L-cysteine levels and ROS and the effects of acetylcysteine on ROS, apoptosis, mitochondrial membrane potential, and apoptosis-related proteins expression disrupted by celastrol in HL-60 cells. (**a**) L-cysteine levels were decreased after celastrol treatment in HL-60 cells. Bar chart shows the statistical results from six independent experiments (*p < 0.05). (**b**) Celastrol induced the accumulation of ROS in HL-60 cells. Bar chart shows the statistical results from three independent experiments. *p < 0.05 when compared with control. (**c**) Acetylcysteine decreased HL-60 cells ROS induced by celastrol. Bar chart shows the statistical results from three independent experiments. Asterisks indicated statistical significance (***p < 0.001). **(d**) Acetylcysteine rescued HL-60 cells from increased apoptosis induced by celastrol. Bar chart shows the statistical results from three independent experiments. Asterisks indicated statistical significance (***p < 0.001). (**e**) Acetylcysteine rescued the decrease of mitochondrial membrane potential induced by celastrol. Bar chart shows the statistical results from three independent experiments. Asterisks indicated statistical significance (***p < 0.001). (**f**) Acetylcysteine inhibited the up-regulation of apoptosis-related proteins induced by celastrol in HL-60 cells. The quantification results of relative protein levels from three independent experiments are shown. Asterisks indicated statistical significance (*p < 0.05).

To verify the change in cysteine metabolism *in vivo*, we analysed L-cysteine in HL-60 cells *in vitro* and found that it was decreased after celastrol treatment in a dose-related manner, as assessed by Spearman correlation analysis (rs = −0.4522, p = 0.0265) (Fig. 3a). As cysteine and its related glutathione metabolism were enriched *in vivo* and the accumulation of intracellular ROS is one of the most important upstream stimuli of p53 activation in apoptosis^29^, the above metabolomics findings prompted us to focus on the intracellular ROS level after celastrol treatment, as ROS might be the intermediate linking the observed deficiency in oxidized glutathione and its upstream metabolites with the decreased anti-oxidative capacity and increased apoptosis in HL-60 cells after celastrol treatment. As shown in Fig. 3b, ROS was detected in control HL-60 cells, and the ROS positive control reagent Rosup led to a dramatic increase in the ROS level in the treated cells, indicating the efficiency of the ROS detection method. The intracellular ROS level was significantly increased in a dose-dependent manner after celastrol treatment (Fig. 3b). Acetylcysteine is an upstream metabolite of cysteine metabolism and was a significantly decreased metabolite after celastrol treatment (Fig. 2). Acetylcysteine exerts an anti-oxidant effect related to its role as a metabolic precursor of glutathione^30^. Based on the metabolomics findings, we used acetylcysteine to disrupt cysteine metabolism in order to verify the connection among cysteine metabolism, ROS and apoptosis. The ROS level in HL-60 cells treated with both celastrol and acetylcysteine was drastically decreased to the level in control cells, indicating that cysteine metabolism plays a key role in the induction of ROS by celastrol (Fig. 3c).

### Acetylcysteine reversed celastrol-induced apoptosis of HL-60 cells

As shown in Fig. 3d, celastrol treatment induced significant apoptosis of HL-60 cells, and acetylcysteine treatment exerted no effect on apoptosis. The apoptosis rate of cells treated with both celastrol and acetylcysteine was drastically decreased to the level of control cells, indicating that cysteine metabolism plays a key role in celastrol-induced apoptosis of HL-60 cells.

### Acetylcysteine reversed the dramatic decrease in the mitochondrial membrane potential and upregulation of apoptosis-related proteins

ROS are mainly produced by mitochondria^31^. To further identify the intermediate linking cysteine metabolism, ROS and apoptosis, we measured the mitochondrial membrane potential. The mitochondrial membrane potential was significantly decreased after celastrol treatment, and this decrease was dramatically reversed by the addition of acetylcysteine, indicating that cysteine metabolism-initiated, ROS-triggered apoptosis might occur through the mitochondrial pathway after celastrol treatment (Fig. 3e). We next assessed the effect of celastrol on the protein levels of p53, Bax, cleaved caspase 9 and cleaved caspase 3. As shown in Figs. 3f, S7–S10, the protein levels of p53, Bax, cleaved caspase 9 and cleaved caspase 3 were significantly increased after celastrol treatment, and this increase was dramatically inhibited in the presence of acetylcysteine, indicating that cysteine metabolism plays a role in celastrol-induced mitochondrial apoptosis in HL-60 cells.

### Cysteine metabolism played a role in celastrol-induced apoptosis of NB-4 cells

**Figure 4:**
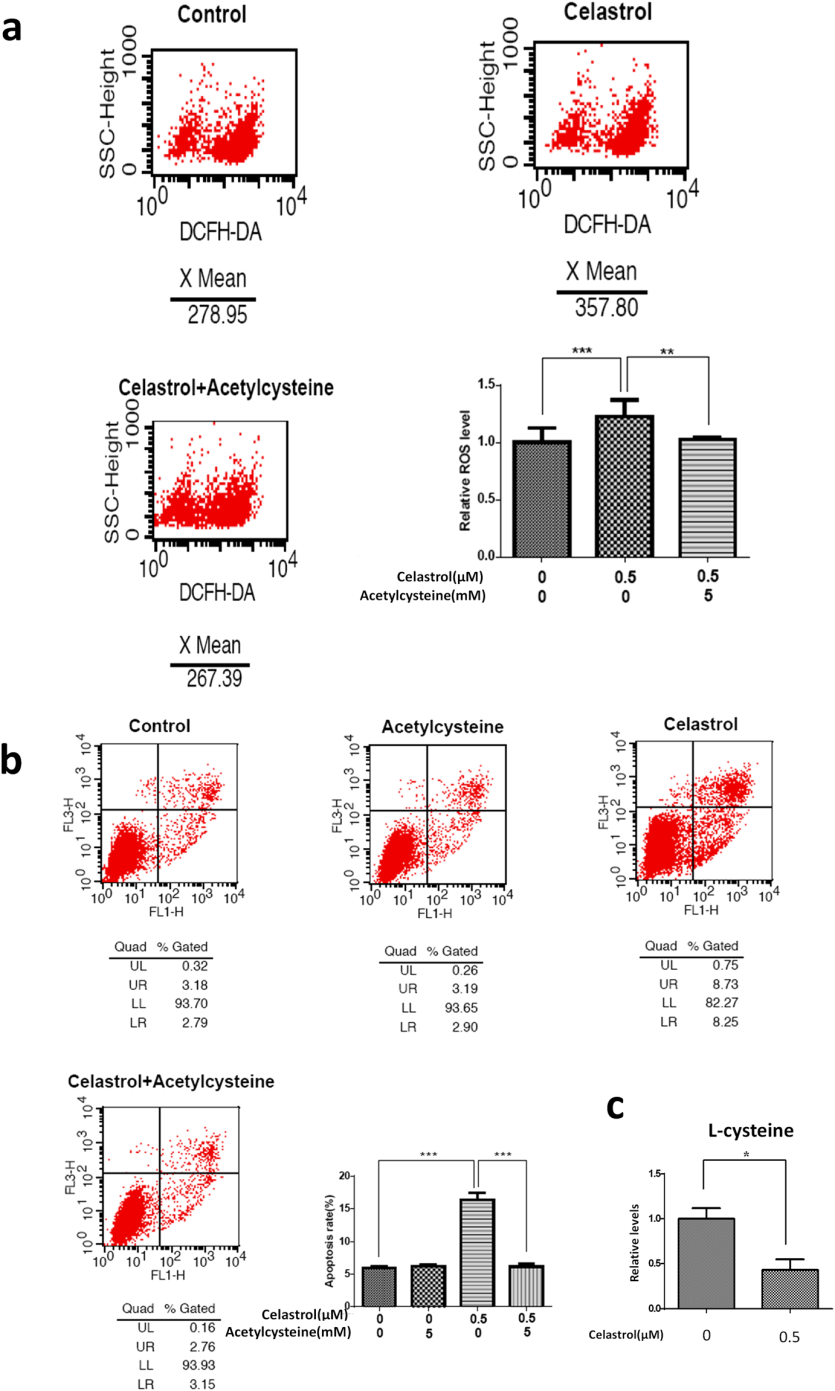
The effects of celastrol on L-cysteine levels and the effect of acetylcysteine on ROS, apoptosis disrupted by celastrol in NB-4 cells. **(a**) Acetylcysteine decreased NB-4 cells ROS induced by celastrol. Bar chart shows the statistical results from three independent experiments. Asterisks indicated statistical significance (**p < 0.01, ***p < 0.001). (**b)** Acetylcysteine rescued NB-4 cells from increased apoptosis induced by celastrol. Bar chart shows the statistical results from three independent experiments. Asterisks indicated statistical significance (***p < 0.001). (**c**) L-cysteine levels were decreased after 0.5 μM celastrol treatment in NB-4 cells. Bar chart shows the statistical results from three independent experiments. Asterisks indicated statistical significance (*p < 0.05).

To determine whether celastrol operated through the same metabolic pathway to induce apoptosis in different model systems, we used human APL NB-4 cells. Similar to its effects on HL-60 cells, celastrol induced ROS accumulation and apoptosis in NB-4 cells; effects were reversed by acetylcysteine (Fig. 4a,b). In addition, L-cysteine, the key metabolite of cysteine metabolism, was significantly decreased after celastrol treatment, validating the changes in cysteine metabolism in different cell models (Fig. 4c).

## Discussion

In the xenograft tumour mouse model, celastrol inhibited tumour growth, induced apoptosis, and upregulated pro-apoptotic proteins (Fig. 1). Unbiased metabolomics profiling revealed that a decrease in the metabolites involved in cysteine metabolism was the key metabolic change after celastrol treatment (Fig. 2). Cysteine metabolism and its derived metabolites are related to cells’ anti-oxidative stress capacity^32^, and the final product of cysteine metabolism is glutathione. Glutathione is an important biological anti-oxidant and can protect important cellular components from ROS-induced damage^33^. ROS are formed by the transfer of one electron from a redox donor to molecular oxygen (O_2_)^31^. Emerging evidence suggests that ROS are critically involved in cancer cell functions^34^. Cancer cells, which have higher levels of basal ROS than normal cells, are likely to be more vulnerable to damage by further ROS insults^35^. Hypothesis-free screening indicated that the decrease in the anti-oxidative capacity and accumulation of intracellular ROS in HL-60 cells were key events after celastrol treatment. The increased ROS level was then verified *in vitro* (Fig. 3).

In this study, we found that the mitochondrial membrane potential significantly decreased after celastrol treatment (Fig. 3). Mitochondria, one of the most important cellular organelles, can produce energy, generate ROS, regulate cell signalling and participate in biosynthetic metabolism^36^. Mitochondria can impart considerable flexibility to tumour cell survival and growth under harsh environmental conditions, such as nutrient depletion, hypoxia, and cancer treatment exposure, and many classical hallmarks of cancer result in altered mitochondrial function. Therefore, mitochondria are key factors in tumourigenesis and treatment.

The tumour suppressor p53 plays a pivotal role in regulating the cell cycle, genomic integrity and apoptosis^37^. Among signalling components, p53 has one of the highest sensitivities to the cellular redox state^38^. p53 mutations often occur in most cancers. However, in the vast majority of AML cells, wild-type p53 is retained^39^. ROS may attack proteins and nucleic acids, and this event is followed by upregulation of wild-type p53. Upregulation of p53 was also verified in this study after celastrol-induced elevation of intracellular ROS levels (Fig. 3). Notably, as a sensor of cellular stress, p53 is a critical initiator of the intrinsic apoptotic pathway under robust or sustained stress^40^.

Apoptosis plays a critical role during various physiological (vertebrate development and postnatal tissue homeostasis) and pathological processes, including tumourigenesis. Resistance to apoptosis is a hallmark of cancer cells^41,42^. Therefore, targeting apoptosis for anticancer therapy is promising. The mitochondrial pathway of apoptosis is activated following cellular stress (DNA damage, nutrient deprivation, endoplasmic reticulum stress, and so on)^43^. The mitochondrial pathway of apoptosis is essential for metazoan development, tissue homeostasis, and cellular responses to therapeutics^43^. In human cancers, signalling via the mitochondrial (intrinsic) pathway of apoptosis is frequently impaired^44^. BCL-2 family proteins, especially Bax, are the central regulators of the intrinsic apoptotic pathway via the control of mitochondrial outer membrane permeabilization (MOMP)^45^, which is frequently the decisive event preceding cell death^46^. In the present study, we verified the pro-apoptotic effect of celastrol on HL-60 cells via the mitochondrial pathway (Fig. 3). We further found the clearance of ROS reversed the upregulation of p53 and the increased apoptosis induced by celastrol (Fig. 3). The elevated ROS is an activating signal for p53-mediated apoptosis in leukemia^35,47^. Therefore, the elevation of ROS induced p53-mediated mitochondrial apoptosis in leukemia cells after celastrol treatment.

Acetylcysteine is an upstream metabolite of cysteine metabolism and was significantly decreased after celastrol treatment (Fig. 2). Acetylcysteine can support cysteine metabolism to increase glutathione production in order to increase the anti-oxidative stress capacity^30^. In this study, when acetylcysteine was added, the mitochondrial membrane potential was dramatically recovered, and the increases in intracellular ROS, p53 expression and apoptosis were inhibited (Fig. 3), verify ing the involvement of a cysteine metabolism/ROS/p53/Bax/caspase 9/caspase 3 pathway in the apoptosis of HL-60 cells after celastrol treatment.

Studying the effect and underlying mechanism in NB-4 cell lines could enhance the significance of the current findings in APL^26^. In this study, the effect of celastrol on cysteine metabolism and the rescue effect of acetylcysteine on celastrol-induced ROS and apoptosis were also verified in NB-4 cells, indicating that celastrol operates through the same metabolic pathway to induce apoptosis in HL-60 and NB-4 cells (Fig. 4).

## Conclusions

**Figure 5:**
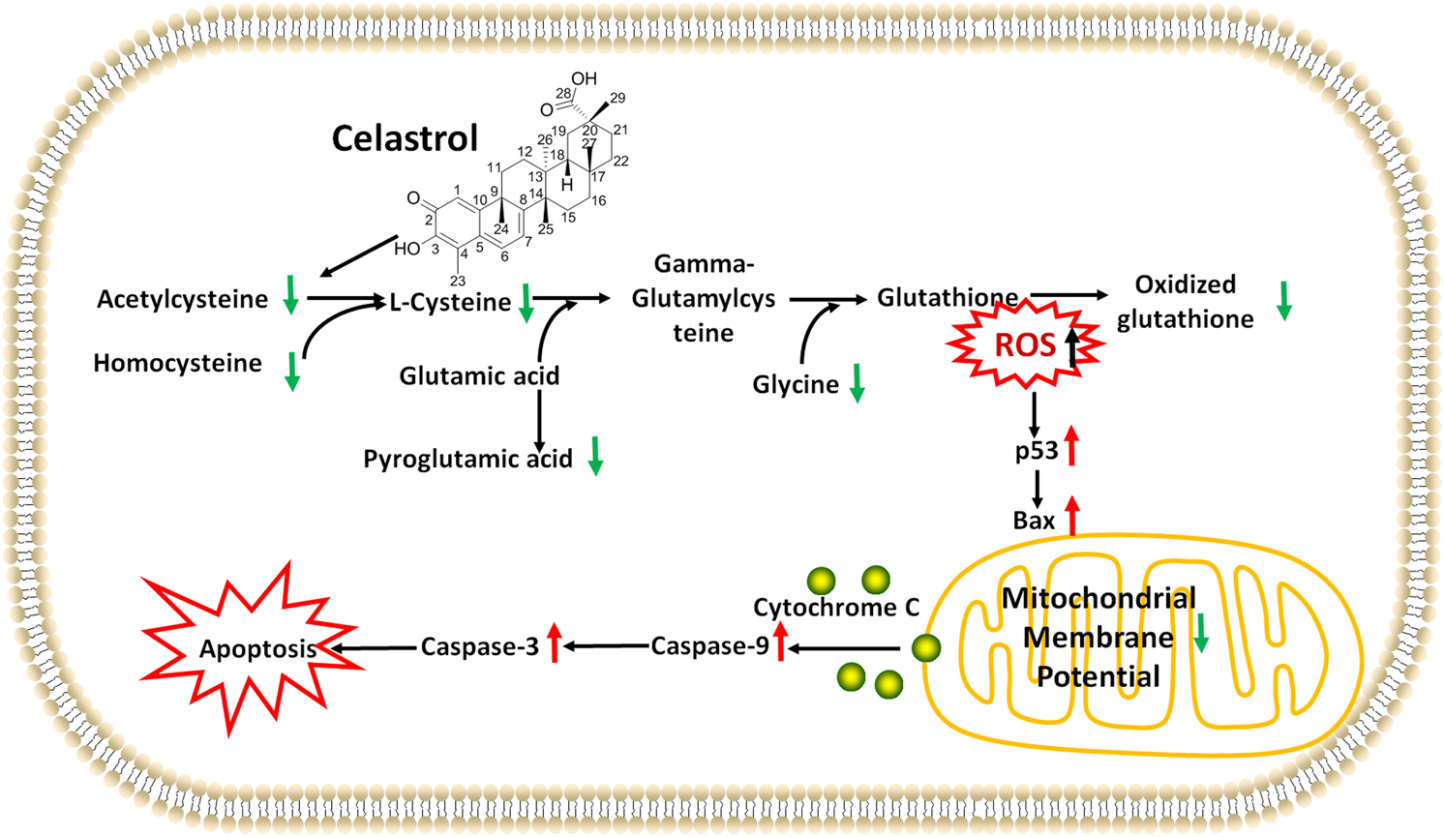
Cysteine metabolism/ROS/p53/Bax/caspase 9/caspase 3 pathway in APL cells after celastrol treatment.

In conclusion, by combining an *in vivo* unbiased metabolomics analysis with follow-up *in vitro* experiments, we found consistent results indicating that cysteine metabolism was the key metabolic change in HL-60 cells and NB-4 cells after celastrol treatment. This finding linked the depleted intracellular anti-oxidative capacity, increased ROS accumulation, and increased mitochondrial apoptosis in human HL-60 and NB-4 cells (Fig. 5), thus providing a novel underlying mechanism through which celastrol might be used to treat AML, especially APL.

## Materials and Methods

### Cell culture

Human HL-60 cells and NB-4 cells were obtained from the Cell Bank of the Chinese Academy of Sciences (Shanghai, China) and grown in Iscove's modified Dulbecco's medium supplemented with 20% foetal bovine serum, 100 IU/ml penicillin and 100 μg/ml streptomycin. Purified celastrol (purity ≥98%, HPLC-grade) and dimethyl sulfoxide (DMSO) were purchased from Sigma-Aldrich (St. Louis, MO, USA). Acetylcysteine (purity >99%) was purchased from Beyotime (Shanghai, China).

### Nude mouse xenograft tumour assay

According to protocols in previous reports^48–50^, after one week of acclimation, male BALB/c nude mice (five weeks old and weighing 20–22 g, obtained from NLARSH (Shanghai, China)) were injected subcutaneously with HL-60 cells (5×10^6^ in 100 μl of PBS) under the shoulder. Tumours were allowed to grow to a volume of 100 to 200 mm^3^. Then, mice were randomized into two groups (9 mice per group), and injected intraperitoneally with vehicle control (5% DMSO in 0.9% saline) or celastrol (2 mg/kg/day). The dosage of celastrol was determined according to previous reports^11,51^. Tumour volume (0.5 × larger diameter × smaller diameter) and body weight were measured. Mice were maintained on a 12:12-h light-dark cycle under specific pathogen-free conditions. Mice were fasted for 12 h before sacrifice on day 21 after celastrol treatment. Tumour weight and volume were measured. TUNEL was performed to identify the apoptotic cells in tumours using an ApopTag® Peroxidase *In Situ* Oligo Ligation Kit (Chemicon International, Inc., Cat. #S7200) according to the manufacturer's instructions. Mice were sacrificed after anaesthetization by intraperitoneal injection of ketamine and diazepam. All animal experiments were performed in accordance with the protocol approved by the Nanjing Medical University Institutional Animal Care and Use Comm ittee.

### Metabolomics analysis

Sample preparation was performed according to our previous report^52^. Homogenized tumour tissues (50 mg) (n = 9 per group) were mixed with 150 μl of water and 600 μl of methanol. Tissues were ultrasonicated for 5 min (power: 60%, pulses: 3/3), and the supernatant was obtained after centrifugation at 20,000 × g for 15 min. After drying, the residue was reconstituted for metabolomics analysis. The extracted samples were subjected to unbiased metabolomics analysis according to protocols in our previous studies^52,53^. Metabolomics analysis was performed on a UPLC Ultimate 3000 system (Dionex, Germering, Germany) coupled to a Q-Exactive Orbitrap mass spectrometer (Thermo Fisher Scientific, Bremen, Germany). The UPLC Ultimate 3000 system was used with a C18 column (Hypersil Gold, 1.9 μm, 100 mm × 2.1 mm; Thermo Fisher Scientific), which was maintained at 40 °C. Mobile phase A was 0.1% formic acid in ultra-pure water, and mobile phase B was acetonitrile acidified with 0.1% formic acid. Chromatographic separation was conducted using a multistep gradient with a flow rate of 0.4 ml/min over 15 min. The UPLC autosampler temperature was set at 4 °C, and the injection volume for each sample was 10 μl. All samples were analysed in a randomized fashion to avoid complications related to the injection order. The Orbitrap mass spectrometer was equipped with a heated electrospray source using full scan analysis (70 to 1050 amu) in both positive and negative ion models simultaneously. For both the positive and negative ion modes, the mass spectrometer parameters were set as follows: spray voltage of 3.5 kV for positive ion model and 2.5 kV for negative ion model, capillary temperature of 300 °C, sheath gas flow of 50 arbitrary units, auxiliary gas flow of 13 arbitrary units, sweep gas flow of 0 arbitrary units, S-Lens RF level of 60, and resolution of 70,000 with an automatic gain control (AGC) target of 3 × 10^6^ charges. The mass spectrometer system was calibrated according to the manufacturer's instructions. Chemical identification was based on the retention time and accurate mass of 270 commercial standards using the author-constructed library. The standard compounds were purchased from SigmaAldrich (St. Louis, MO), Damasbeta Co., Ltd. (Basel, Switzerland), Adamas Reagent Co., Ltd. (Shanghai, China), and Aladdin Reagent Company (Shanghai, China). Quality control (QC) sample was prepared by mixing each original sample according to a previous metabolomics study^54^, and was analysed in parallel with original samples.

### Targeted L-cysteine analysis

HL-60 cells and NB-4 cells were plated at a density of 2 × 10^5^ cells/well in six-well plates and exposed to celastrol for 24 h. Cells were harvested and washed three times with ice-cold PBS. Then, 1 ml of ice-cold 50% methanol was added, and all cells were lysed by ultrasonication for 1 min (power: 60%, pulses: 3/3). Following centrifugation (16,000 × g, 10 min, 4 °C), the supernatant was used for detection. L-cysteine was detected using the UPLC Ultimate 3000 system coupled to the Orbitrap mass spectrometer. The conditions of the chromatograph and mass spectrometer were the same as those described in the “Metabolomics analysis” section. The [M+H]+ ions of L-cysteine at m/z 122.02703 were monitored.

### Measurement of intracellular ROS

Intracellular ROS generation was measured by a ROS assay with DCFH-DA (Beyotime, Shanghai, China) according to a previous report^55^. Rosup was used as the positive control reagent according to the manufacturer's instructions. In brief, HL-60 cells were plated at a density of 2 × 10^5^ cells/well in six-well plates and exposed to celastrol (0.125, 0.25 and 0.5 μM) for 24 h. Then, cells were incubated with DCFH-DA (10 μM) for 30 min at 37 °C. DCFH-DA is hydrolysed by intracellular esterase to produce a nonfluorescent DCFH product, that can then be oxidized by ROS to produce a highly fluorescent DCF product. For the follow-up study, HL-60 cells and NB-4 cells were treated with 0.1% DMSO, celastrol (0.5 μM) and celastrol (0.5 μM)+acetylcysteine (5 mM) for 24 h. The level of ROS was measured by flow cytometry (BD Biosciences, NJ, USA). All experiments were repeated at least three times.

### Determination of apoptosis by flow cytometric analysis

HL-60 and NB-4 cells were treated with 0.1% DMSO, acetylcysteine (5 mM), celastrol (0.5 μM) and celastrol (0.5 μM)+acetylcysteine (5 mM) for 24 h. The concentration of acetylcysteine was determined according to a previous report^56^. The percentage of apoptotic cells was assessed using a FITC Annexin V Apoptosis Detection Kit I (BD Biosciences, NJ, USA) as described in the manufacturer's instructions.

### Measurement of the mitochondrial membrane potential

The mitochondrial membrane potential was measured with a JC-1 Mitochondrial Membrane Potential Assay Kit (Yeasen, Shanghai, China) according to the manufacturer's instructions, as described previously^57^. In brief, HL-60 cells were treated with 0.1% DMSO, acetylcysteine (5 mM), celastrol (0.5 μM) and celastrol (0.5 μM) + acetylcysteine (5 mM) for 24 h. Then, cells were incubated with JC-1 for 20 min in the dark at 37 °C. After being washed twice with staining buffer, cells were analysed by flow cytometry (BD Biosciences, NJ, USA). JC-1 forms aggregates and emits red fluorescence to indicate a normal mitochondrial membrane potential. This red fluorescence was collected as the PE signal, while the accumulation of green fluorescent monomers due to depolarization of the mitochondrial membrane potential was indicated by an increase in FITC fluorescence.

### Western blot analysis

Western blot analysis was performed as described previously^58^ using the following antibodies: primary rabbit polyclonal antibodies against cleaved caspase 9 (Cat. #9505T), cleaved caspase 3 (Cat. #9664T), Bax (Cat. #2772), and p53 (Cat. #2524) (Cell Signaling Technology, Beverly, MA, USA); a mouse monoclonal antibody against β-actin (Cat. #AA128, Beyotime, Shanghai, China); and an HRP-conjugated secondary antibody (Cat. #A0208, Cat #A0216, Beyotime, Shanghai, China). Western blot data were quantified with Image-Pro Plus software. All experiments were repeated at least three times.

### Statistical analyses

Statistical analysis of the data was performed using the Stata statistical package (Version 9.2, Stata Corp, LP); “R” (http://cran.r-project.org/), a freely available open source software package; and SIMCA-P software (Umetrics, Sweden). The metabolomics data were imported into SIMCA-P software for multivariate analysis. All data were UV-scaled and automatically log transformed by the software. PCA was applied to identify the metabolic changes between groups. The differences in metabolomics data between the treatment group and control group were identified with t-tests. To improve the statistical robustness, we used the FDR correction for multiple testing. We imported the changed metabolites into the “enrichment analysis” module to analyse the key metabolic changes (www.metaboanalyst.ca/). Statistically significant differences between two groups and among more than two groups were determined using a two-sided, unpaired t-test and one-way ANOVA with Dunnett's post hoc test, respectively. Values are expressed as the means ± standard errors. A *p* value of <0.05 was considered statistically significant.

## Supplementary information

Supplementary material.

## Data Availability

The raw metabolomic data are available in the electronic Supplementary Material.
