# Supplementary material for: Metabolomics Reveals that Cysteine Metabolism Plays a Role in Celastrol-Induced Mitochondrial Apoptosis in HL-60 and NB-4 Cells
Source: Sci Rep. 2020 Jan 16;10:471. doi: 10.1038/s41598-019-57312-y (PMC6965619; doi:10.1038/s41598-019-57312-y)
Supplement: Supplementary file 1 — Supplementary material. [file 41598_2019_57312_MOESM1_ESM.docx]

Metabolomics Reveals that Cysteine Metabolism Plays a Role in Celastrol-Induced Mitochondrial Apoptosis in HL-60 and NB-4 Cells

Minjian Chen^1,2,3^, Jing Yang^4^ , Lei Li^5^ , Yanhui Hu^2,6^, Xiaomei Lu^5^, Rongli Sun^7^, Yubang Wang^2,6^, Xinru Wang^1,2^ and Xiaoling Zhang^5*^

^1^ State Key Laboratory of Reproductive Medicine, Center for Global Health, School of Public Health, Nanjing Medical University, Nanjing, 211166, China

^2^ Key Laboratory of Modern Toxicology of Ministry of Education, Nanjing Medical University, Nanjing, 211166, China

^3^ Wuxi Maternal and Child Health Hospital Affiliated to Nanjing Medical University, Wuxi, 214002, China

^4^ Experiment Center for Teaching and Learning, Shanghai University of Traditional Chinese Medicine, Shanghai, 201203, China

^5^ Department of Hygienic Analysis and Detection, Nanjing Medical University, Nanjing, 211166, China

^6^ Safety Assessment and Research Center for Drug, Pesticide, and Veterinary Drug of Jiangsu Province, School of Public Health, Nanjing Medical University, Nanjing 211166, China

^7^ Key Laboratory of Environmental Medicine Engineering, Ministry of Education, School of Public Health, Southeast University, Nanjing, 210009, China

***** Correspondence: zhangxl3@njmu.edu.cn; Tel.: +86 2586868402

Fig.S1 The chemical structure of celastrol.


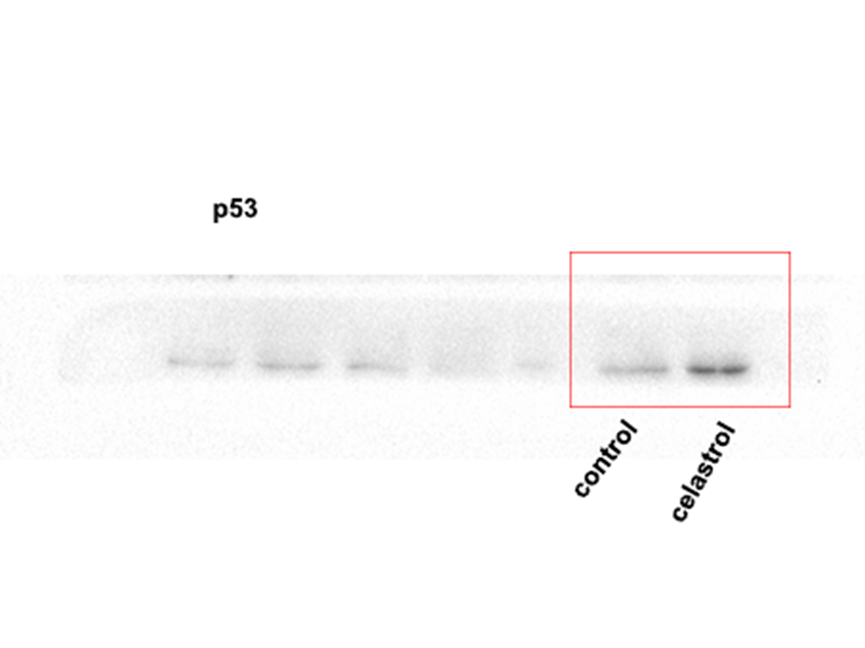


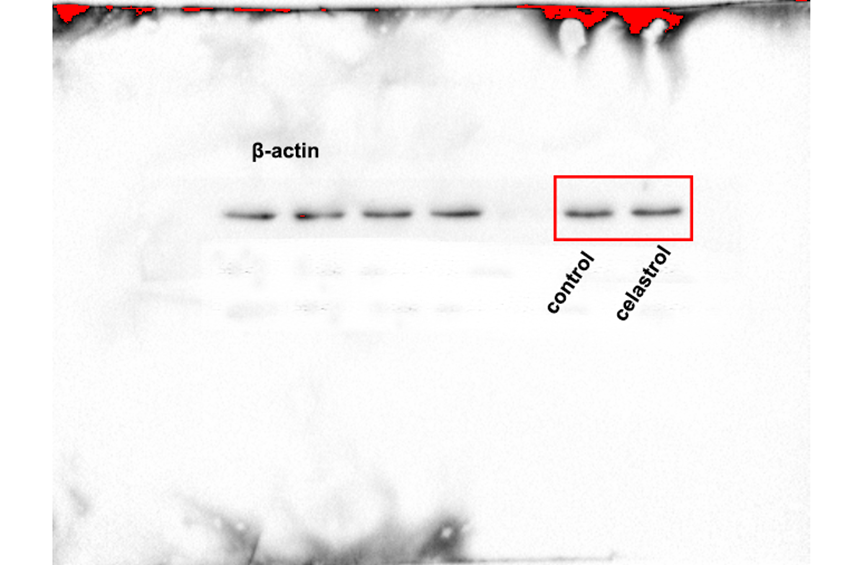


Fig.S2 Uncropped images of the original western blots of p53 and corresponding loading control for Fig.1e.


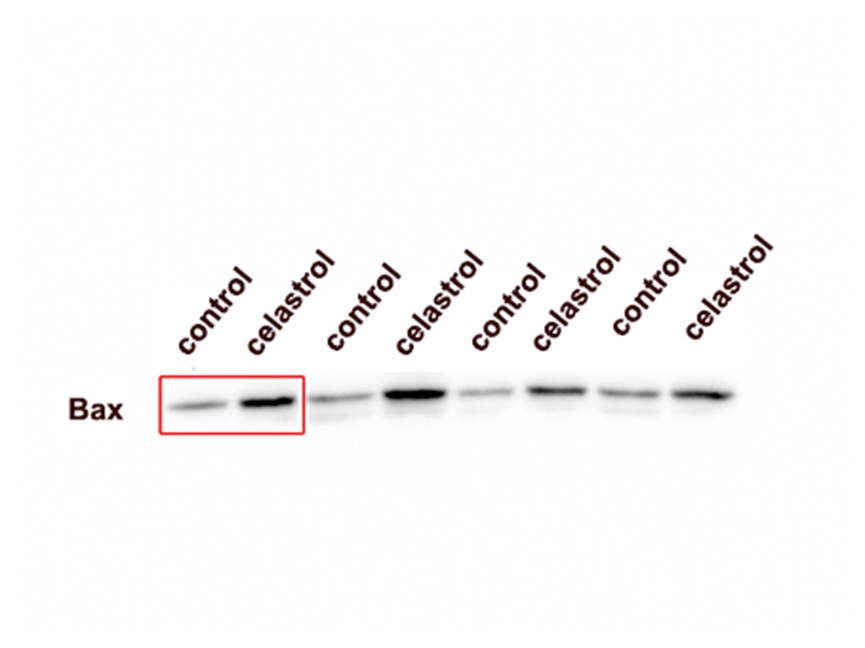


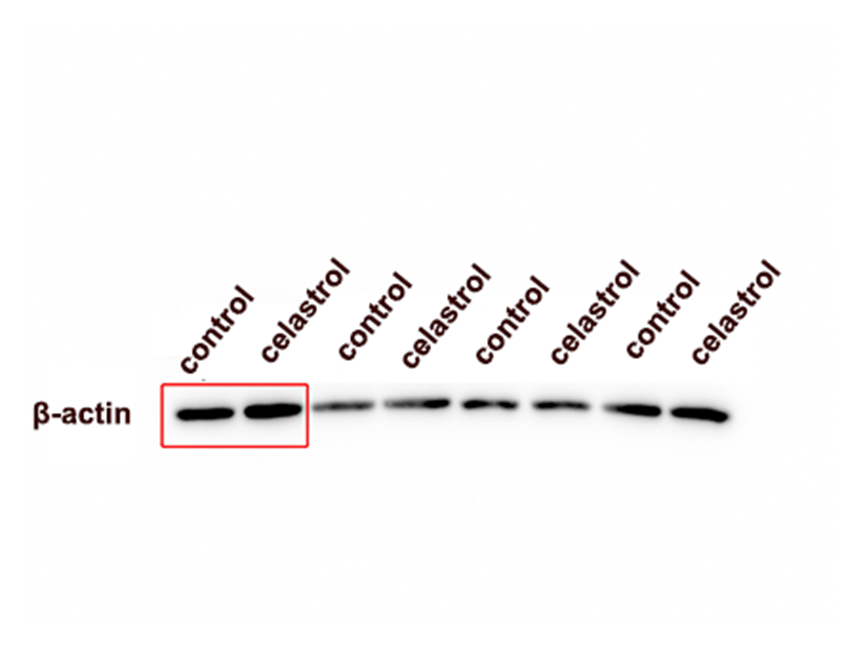


Fig.S3 Uncropped images of the original western blots of Bax and corresponding loading control for Fig.1e.


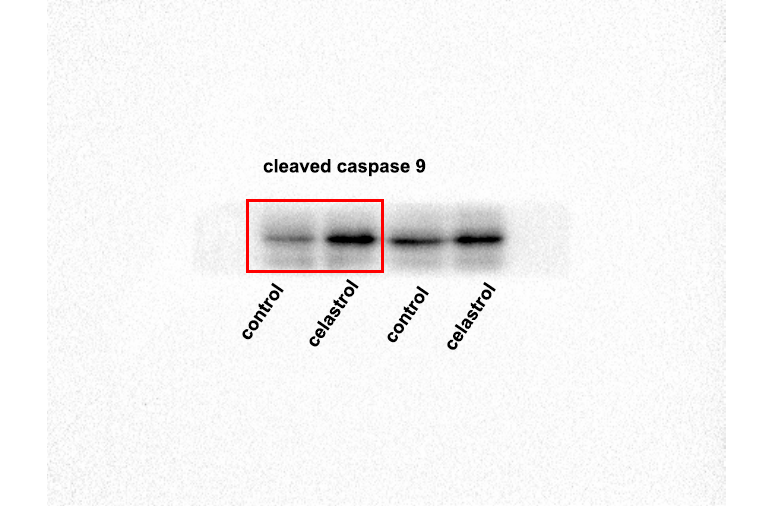


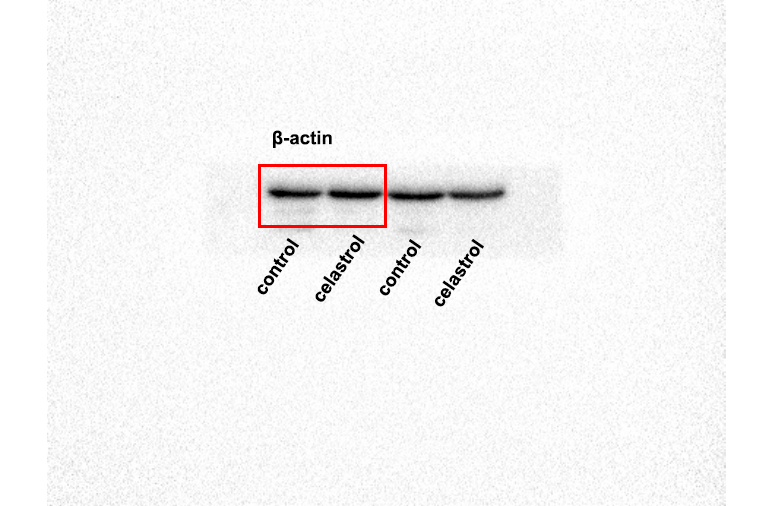


Fig.S4 Uncropped images of the original western blots of cleaved caspase 9 and corresponding loading control for Fig.1e.


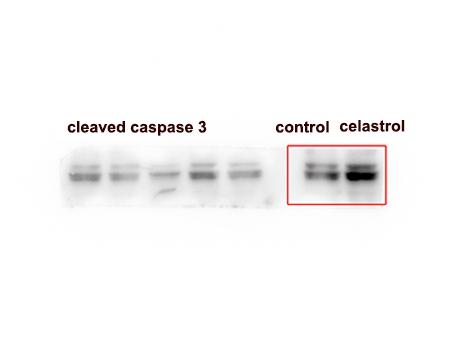

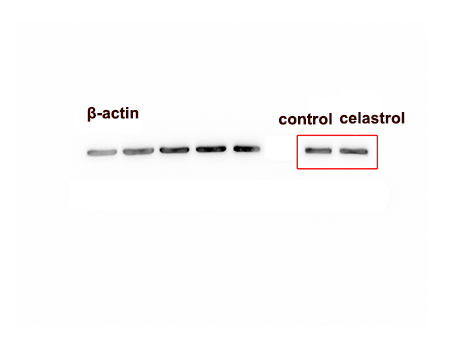


Fig.S5 Uncropped images of the original western blots of cleaved caspase 3 and corresponding loading control for Fig.1e.


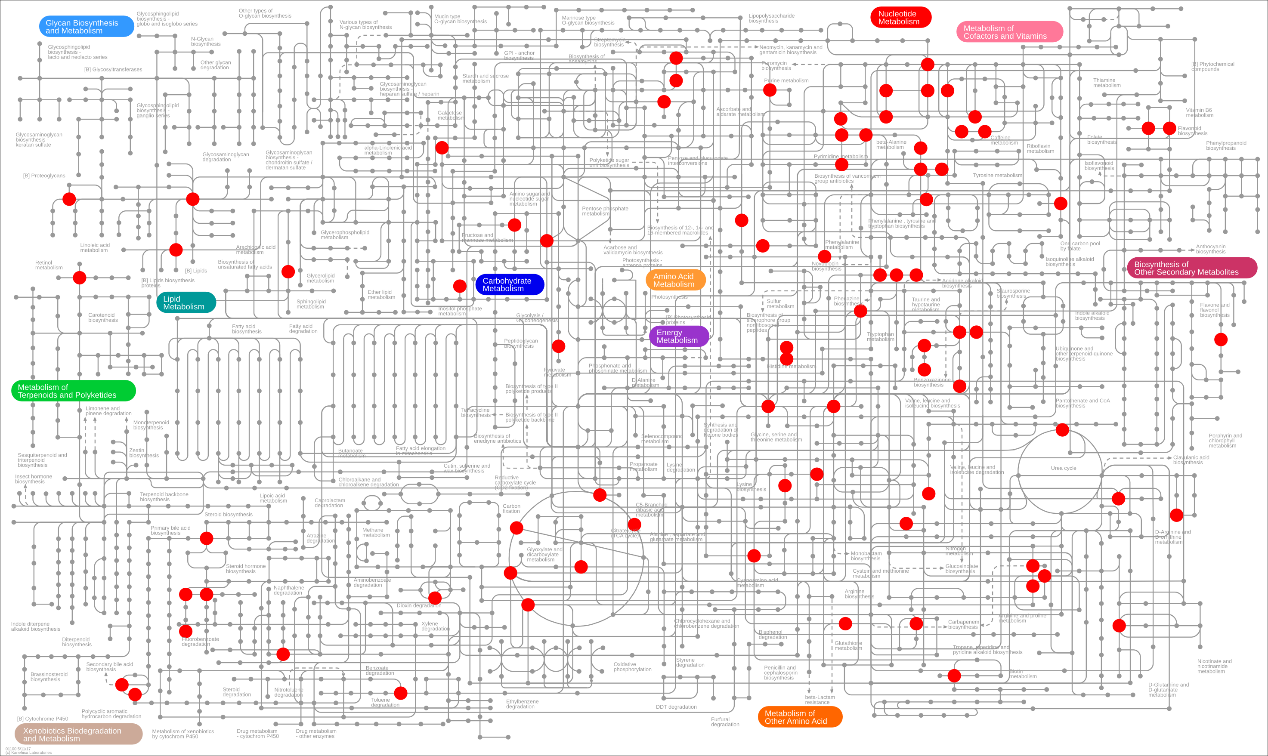


Fig.S6 The detected metabolites in KEGG general metabolic pathway map^1^. The detected metabolites not indicated in the general pathway map are not shown. The original general metabolic pathway map is available at https://pathways.embl.de/ipath3.cgi.


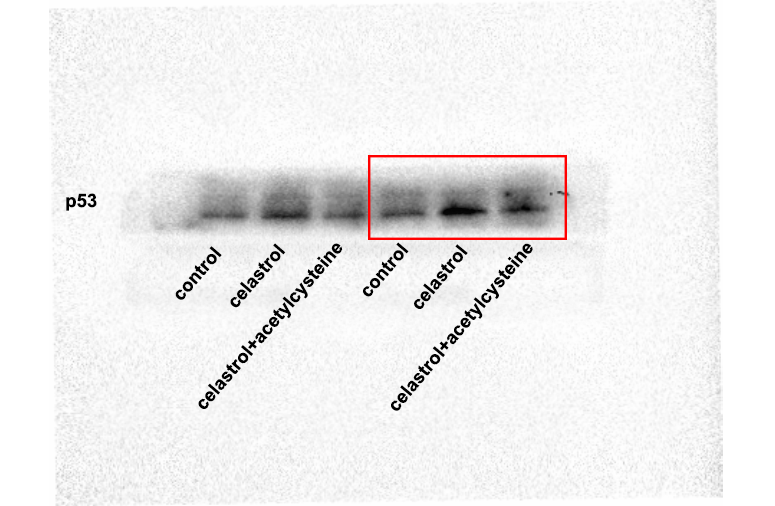


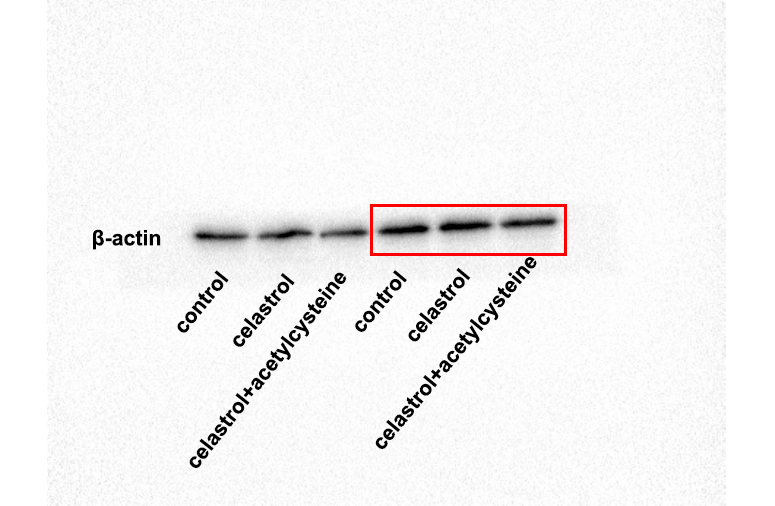


Fig.S7 Uncropped images of the original western blots of p53 and corresponding loading control for Fig.3f.


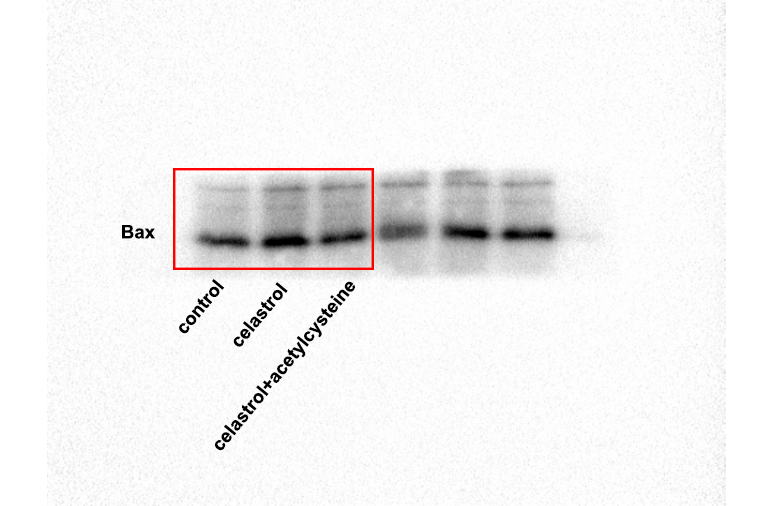


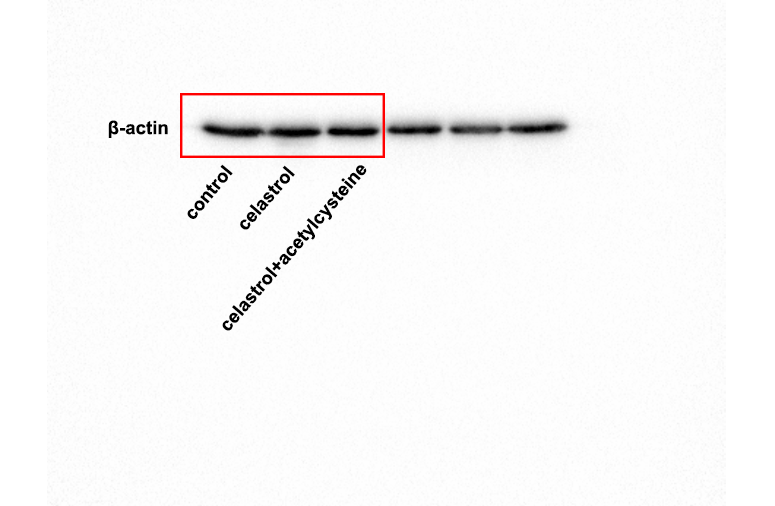


Fig.S8 Uncropped images of the original western blots of Bax and corresponding loading control for Fig.3f.


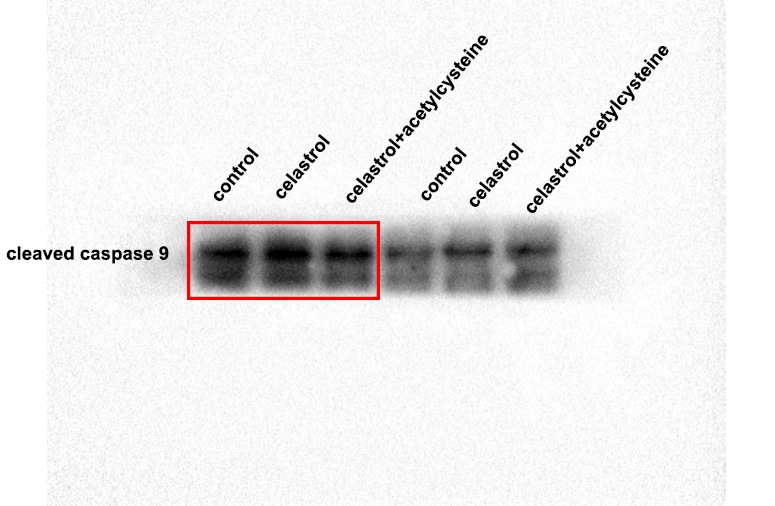


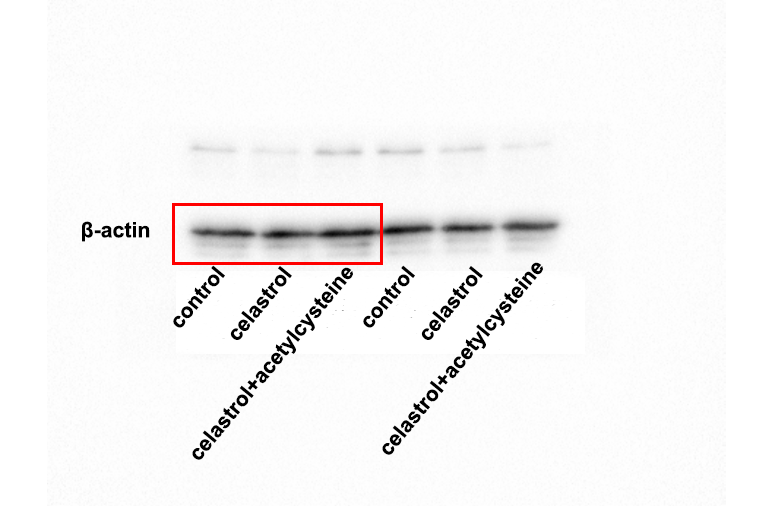


Fig.S9 Uncropped images of the original western blots of cleaved caspase 9 and corresponding loading control for Fig.3f.


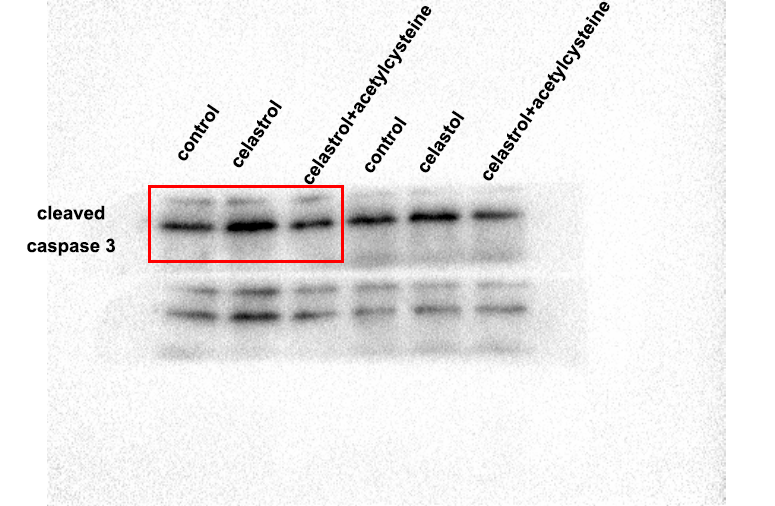


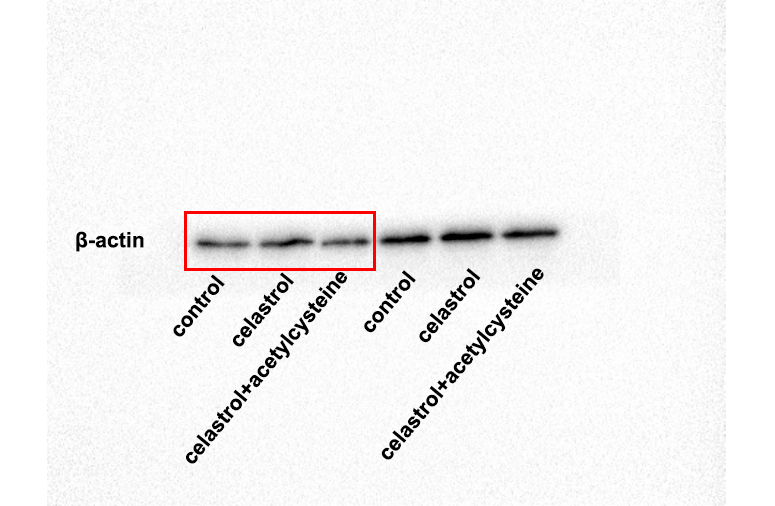


Fig.S10 Uncropped images of the original western blots of cleaved caspase 3 and corresponding loading control for Fig.3f.

Table S1 Metabolomics analysis of tumour tissue treated with celastrol in vivo.

| Metabolite | Fold of control | p | FDR q value |
| --- | --- | --- | --- |
| 17-Hydroxyprogesterone | 1.77 | 8.40E-04 | 1.67E-03 |
| 2-Methoxyestrone | 0.86 | 6.27E-05 | 2.07E-04 |
| 3-Indoleacetonitrile | 1.18 | 1.50E-05 | 7.42E-05 |
| 3-Indolepropionic acid | 6.83 | 1.08E-01 | 1.12E-01 |
| 3-Methyladenine | 0.39 | 4.35E-03 | 6.68E-03 |
| 3-Methylhistidine | 0.87 | 3.03E-09 | 5.58E-08 |
| 3-Methylxanthine | 1.08 | 9.03E-05 | 2.71E-04 |
| 3-Pyridylacetic acid | 1.69 | 9.43E-04 | 1.84E-03 |
| 4-Hydroxyproline | 0.08 | 1.09E-04 | 3.06E-04 |
| 4-Methylcatechol | 1.43 | 1.76E-06 | 1.20E-05 |
| 5-Hydroxylysine | 0.47 | 9.44E-06 | 4.87E-05 |
| 5-Methoxytryptamine | 0.41 | 1.14E-11 | 2.94E-10 |
| 5'-Methylthioadenosine | 0.14 | 6.72E-03 | 9.86E-03 |
| 7b-Hydroxycholesterol | 1.18 | 3.08E-02 | 3.46E-02 |
| Acetylcysteine | 0.38 | 9.24E-03 | 1.27E-02 |
| Acetylglycine | 3.21 | 2.76E-03 | 4.50E-03 |
| Adenine | 0.32 | 6.18E-253 | 7.98E-251 |
| Adenosine | 19.80 | 1.65E-01 | 1.69E-01 |
| Allantoin | 2.78 | 1.13E-08 | 1.62E-07 |
| AMP | 0.04 | 3.62E-02 | 3.99E-02 |
| Arachidonic acid | 0.03 | 4.65E-02 | 4.96E-02 |
| Argininosuccinic acid | 0.64 | 2.58E-07 | 2.22E-06 |
| Ascorbic acid | 0.11 | 3.37E-04 | 7.63E-04 |
| Beta-Alanine | 0.43 | 1.02E-04 | 2.99E-04 |
| Bilirubin | 0.19 | 7.12E-05 | 2.24E-04 |
| Biotin | 0.59 | 7.57E-04 | 1.53E-03 |
| Carnosine | 0.41 | 1.93E-03 | 3.23E-03 |
| Cholesterol | 1.09 | 3.60E-07 | 2.91E-06 |
| Cholic acid | 7.09 | 1.01E-03 | 1.94E-03 |
| cis-Aconitic acid | 0.84 | 2.60E-04 | 6.38E-04 |
| Citric acid | 1.17 | 4.40E-04 | 9.46E-04 |
| Cortisone | 8.29 | 3.44E-03 | 5.47E-03 |
| Creatinine | 1.05 | 4.04E-04 | 8.84E-04 |
| Cyclic AMP | 0.10 | 2.63E-02 | 3.01E-02 |
| Cytidine | 0.67 | 2.46E-02 | 2.86E-02 |
| Deoxycytidine | 1.06 | 4.93E-02 | 5.21E-02 |
| Deoxyinosine | 0.02 | 1.79E-03 | 3.08E-03 |
| D-Glucaric acid | 0.09 | 2.91E-21 | 1.25E-19 |
| D-Glucuronic acid | 0.45 | 1.88E-05 | 8.09E-05 |
| D-Glucurono-6,3-lactone | 22.54 | 1.76E-03 | 3.07E-03 |
| Dihydroxyacetone phosphate | 0.97 | 1.41E-09 | 3.04E-08 |
| Dodecanedioic acid | 1.06 | 1.16E-04 | 3.18E-04 |
| Fumaric acid | 0.21 | 1.31E-02 | 1.71E-02 |
| Gluconolactone | 0.27 | 1.49E-03 | 2.67E-03 |
| Glucosamine 6-phosphate | 0.00 | 1.94E-08 | 2.28E-07 |
| Glucose 6-phosphate | 0.01 | 9.67E-08 | 8.91E-07 |
| Glutamic acid | 0.01 | 7.07E-06 | 3.80E-05 |
| Glyceraldehyde | 10.27 | 1.49E-06 | 1.07E-05 |
| Glycerophosphocholine | 0.24 | 1.86E-05 | 8.09E-05 |
| Glycine | 0.74 | 3.80E-05 | 1.44E-04 |
| Glycolic acid | 13.48 | 7.62E-03 | 1.08E-02 |
| Guanine | 0.49 | 2.73E-01 | 2.75E-01 |
| Hexadecanedioic acid | 5.62 | 7.25E-04 | 1.49E-03 |
| Hippuric acid | 4.01 | 1.38E-02 | 1.76E-02 |
| Histamine | 1.25 | 3.61E-02 | 3.99E-02 |
| Homocysteine | 0.35 | 4.77E-05 | 1.69E-04 |
| Hypotaurine | 0.57 | 2.68E-06 | 1.64E-05 |
| Indoleacetic acid | 8.94 | 1.50E-02 | 1.90E-02 |
| Inosine | 0.22 | 6.95E-03 | 1.01E-02 |
| Inosinic acid | 0.00 | 1.55E-02 | 1.94E-02 |
| Itaconic acid | 0.50 | 6.05E-05 | 2.05E-04 |
| L-Acetylcarnitine | 0.29 | 5.66E-04 | 1.20E-03 |
| L-Aspartyl-L-phenylalanine | 0.04 | 3.48E-03 | 5.47E-03 |
| L-Carnitine | 0.29 | 3.97E-04 | 8.83E-04 |
| L-Cysteine | 0.04 | 4.03E-250 | 2.60E-248 |
| L-Cystine | 0.09 | 4.12E-02 | 4.50E-02 |
| Levulinic acid | 0.75 | 4.24E-05 | 1.56E-04 |
| L-Histidine | 1.09 | 1.64E-05 | 7.79E-05 |
| Linoleic acid | 0.32 | 1.37E-03 | 2.56E-03 |
| L-Leucine | 0.56 | 4.13E-03 | 6.42E-03 |
| L-Lysine | 0.56 | 3.43E-05 | 1.38E-04 |
| L-Malic acid | 0.45 | 2.62E-04 | 6.38E-04 |
| L-Palmitoylcarnitine | 0.49 | 1.13E-01 | 1.17E-01 |
| L-Phenylalanine | 0.80 | 2.74E-02 | 3.10E-02 |
| L-Proline | 0.06 | 3.67E-05 | 1.43E-04 |
| L-Serine | 2.54 | 4.92E-08 | 4.88E-07 |
| L-Tryptophan | 1.84 | 2.92E-04 | 6.92E-04 |
| Maleic acid | 0.82 | 2.38E-04 | 6.14E-04 |
| Melatonin | 2.08 | 1.18E-02 | 1.58E-02 |
| N-Acetylglutamic acid | 0.26 | 1.30E-02 | 1.71E-02 |
| N-Acetylglutamine | 1.37 | 6.36E-03 | 9.43E-03 |
| N-Acetylleucine | 4.73 | 1.56E-02 | 1.94E-02 |
| N-Acetyl-L-methionine | 0.13 | 1.65E-03 | 2.92E-03 |
| N-Acetyl-L-phenylalanine | 50.91 | 1.36E-02 | 1.76E-02 |
| N-Acetyl-L-tyrosine | 27.98 | 3.28E-01 | 3.28E-01 |
| N-Acetylneuraminic acid | 0.10 | 1.69E-05 | 7.79E-05 |
| N-Acetylvaline | 4.72 | 2.12E-02 | 2.54E-02 |
| N-Alpha-acetyllysine | 0.64 | 2.95E-04 | 6.92E-04 |
| N-Formyl-L-methionine | 0.30 | 1.70E-02 | 2.06E-02 |
| Niacinamide | 0.07 | 1.12E-03 | 2.13E-03 |
| N-Oleoylethanolamine | 0.06 | 1.93E-01 | 1.96E-01 |
| Norvaline | 1.11 | 4.63E-03 | 7.03E-03 |
| Octadecanamide | 0.13 | 4.29E-02 | 4.65E-02 |
| Orotic acid | 0.22 | 1.12E-08 | 1.62E-07 |
| Oxidized glutathione | 0.87 | 3.14E-03 | 5.07E-03 |
| Pantothenol | 0.83 | 4.47E-02 | 4.81E-02 |
| Petroselinic acid | 1.76 | 1.07E-04 | 3.06E-04 |
| Pipecolic acid | 0.54 | 1.44E-06 | 1.07E-05 |
| Progesterone | 0.86 | 1.71E-02 | 2.06E-02 |
| Prostaglandin E2 | 0.03 | 3.51E-08 | 3.77E-07 |
| Purine | 0.05 | 1.55E-08 | 2.00E-07 |
| Pyridoxal | 0.52 | 1.84E-03 | 3.13E-03 |
| Pyridoxal 5'-phosphate | 0.22 | 1.38E-04 | 3.71E-04 |
| Pyroglutamic acid | 0.18 | 1.44E-03 | 2.62E-03 |
| Retinal | 0.94 | 3.04E-05 | 1.27E-04 |
| Rhamnose | 5.72 | 2.10E-04 | 5.53E-04 |
| Riboflavin | 0.46 | 8.98E-03 | 1.25E-02 |
| Ribothymidine | 0.04 | 3.11E-06 | 1.82E-05 |
| Salicylic acid | 1.46 | 2.55E-04 | 6.38E-04 |
| Sebacic acid | 0.94 | 6.42E-05 | 2.07E-04 |
| Serotonin | 186.11 | 1.40E-03 | 2.59E-03 |
| Sorbitol | 0.40 | 1.14E-02 | 1.55E-02 |
| Sphingosine | 0.01 | 5.79E-03 | 8.68E-03 |
| Succinic acid | 2.34 | 6.76E-04 | 1.41E-03 |
| Taurine | 0.20 | 2.54E-06 | 1.64E-05 |
| Taurocholic Acid | 10.58 | 8.80E-02 | 9.23E-02 |
| Tetradecanedioic acid | 0.23 | 2.43E-02 | 2.85E-02 |
| Theophylline | 1.51 | 1.28E-02 | 1.70E-02 |
| Thymine | 0.07 | 4.85E-05 | 1.69E-04 |
| Thyroxine | 5.55 | 2.34E-02 | 2.77E-02 |
| Tryptamine | 1.29 | 3.82E-06 | 2.14E-05 |
| Tryptophanol | 1.36 | 7.03E-03 | 1.01E-02 |
| Uracil | 0.11 | 3.05E-04 | 7.03E-04 |
| Urea | 9.90 | 8.04E-03 | 1.13E-02 |
| Ureidopropionic acid | 3.20 | 2.37E-03 | 3.92E-03 |
| Uridine | 0.14 | 7.74E-05 | 2.38E-04 |
| Valeric acid | 1.03 | 5.41E-13 | 1.75E-11 |
| Xanthosine | 0.11 | 2.63E-02 | 3.01E-02 |
| Xanthurenic acid | 11.63 | 1.70E-02 | 2.06E-02 |

Table S2 Pathway enrichment of metabolomics analysis of tumour tissue treated with celastrol in vivo.

| Pathway | Total | Expected | Hits | p |
| --- | --- | --- | --- | --- |
| Protein biosynthesis | 19 | 2.79 | 7 | 1.35E-02 |
| Glutathione metabolism | 10 | 1.47 | 4 | 4.56E-02 |
| Taurine and hypotaurine metabolism | 7 | 1.03 | 3 | 6.91E-02 |
| Biotin metabolism | 4 | 0.59 | 2 | 1.05E-01 |
| Beta-alanine metabolism | 13 | 1.91 | 4 | 1.09E-01 |
| Excitatory neural signalling through 5-htr 4 and serotonin | 5 | 0.73 | 2 | 1.59E-01 |
| Intracellular signalling through histamine h2 receptor and histamine | 5 | 0.73 | 2 | 1.59E-01 |
| Histidine metabolism | 11 | 1.62 | 3 | 2.11E-01 |
| Citric acid cycle | 23 | 3.38 | 5 | 2.40E-01 |
| Aspartate metabolism | 12 | 1.76 | 3 | 2.53E-01 |
| Pyrimidine metabolism | 36 | 5.29 | 7 | 2.68E-01 |
| Methionine metabolism | 24 | 3.52 | 5 | 2.69E-01 |
| Malate-aspartate shuttle | 8 | 1.17 | 2 | 3.33E-01 |
| Tryptophan metabolism | 34 | 4.99 | 6 | 3.82E-01 |
| Mitochondrial electron transport chain | 15 | 2.2 | 3 | 3.82E-01 |
| Alpha linolenic acid and linoleic acid metabolism | 9 | 1.32 | 2 | 3.90E-01 |
| Vitamin b6 metabolism | 10 | 1.47 | 2 | 4.45E-01 |
| Pantothenate and coa biosynthesis | 10 | 1.47 | 2 | 4.45E-01 |
| Intracellular signalling through fsh receptor and follicle stimulating hormone | 4 | 0.59 | 1 | 4.71E-01 |
| Ammonia recycling | 18 | 2.64 | 3 | 5.07E-01 |
| Glutamate metabolism | 18 | 2.64 | 3 | 5.07E-01 |
| Glycine, serine and threonine metabolism | 26 | 3.82 | 4 | 5.46E-01 |
| Arginine and proline metabolism | 26 | 3.82 | 4 | 5.46E-01 |
| Corticotropin activation of cortisol production | 5 | 0.73 | 1 | 5.49E-01 |
| Vasopressin regulation of water homeostasis | 5 | 0.73 | 1 | 5.49E-01 |
| Intracellular signalling through pgd2 receptor and prostaglandin d2 | 5 | 0.73 | 1 | 5.49E-01 |
| Urea cycle | 20 | 2.94 | 3 | 5.82E-01 |
| Phenylalanine and tyrosine metabolism | 13 | 1.91 | 2 | 5.91E-01 |
| Glycerolipid metabolism | 13 | 1.91 | 2 | 5.91E-01 |
| Intracellular signalling through prostacyclin receptor and prostacyclin | 6 | 0.88 | 1 | 6.16E-01 |
| Oxidation of branched chain fatty acids | 14 | 2.06 | 2 | 6.33E-01 |
| Beta oxidation of very long chain fatty acids | 14 | 2.06 | 2 | 6.33E-01 |
| Starch and sucrose metabolism | 14 | 2.06 | 2 | 6.33E-01 |
| Purine metabolism | 45 | 6.61 | 6 | 6.71E-01 |
| Sphingolipid metabolism | 15 | 2.2 | 2 | 6.72E-01 |
| Amino sugar metabolism | 15 | 2.2 | 2 | 6.72E-01 |
| Intracellular signalling through adenosine receptor a2a and adenosine | 7 | 1.03 | 1 | 6.72E-01 |
| Steroidogenesis | 32 | 4.7 | 4 | 7.17E-01 |
| Cysteine metabolism | 8 | 1.17 | 1 | 7.21E-01 |
| Glycerol phosphate shuttle | 8 | 1.17 | 1 | 7.21E-01 |
| Nucleotide sugars metabolism | 9 | 1.32 | 1 | 7.62E-01 |
| Riboflavin metabolism | 9 | 1.32 | 1 | 7.62E-01 |
| Pentose phosphate pathway | 18 | 2.64 | 2 | 7.68E-01 |
| Fructose and mannose degradation | 18 | 2.64 | 2 | 7.68E-01 |
| Gluconeogenesis | 27 | 3.96 | 3 | 7.84E-01 |
| Inositol metabolism | 19 | 2.79 | 2 | 7.94E-01 |
| Betaine metabolism | 10 | 1.47 | 1 | 7.98E-01 |
| Glycolysis | 21 | 3.08 | 2 | 8.39E-01 |
| Caffeine metabolism | 12 | 1.76 | 1 | 8.53E-01 |
| Glucose-alanine cycle | 12 | 1.76 | 1 | 8.53E-01 |
| Porphyrin metabolism | 22 | 3.23 | 2 | 8.58E-01 |
| Lysine degradation | 13 | 1.91 | 1 | 8.75E-01 |
| Nicotinate and nicotinamide metabolism | 13 | 1.91 | 1 | 8.75E-01 |
| Galactose metabolism | 25 | 3.67 | 2 | 9.03E-01 |
| Androgen and estrogen metabolism | 17 | 2.5 | 1 | 9.35E-01 |
| Propanoate metabolism | 18 | 2.64 | 1 | 9.44E-01 |
| Retinol metabolism | 18 | 2.64 | 1 | 9.44E-01 |
| Bile acid biosynthesis | 49 | 7.2 | 4 | 9.48E-01 |
| Phospholipid biosynthesis | 19 | 2.79 | 1 | 9.53E-01 |
| Insulin signalling | 19 | 2.79 | 1 | 9.53E-01 |
| Pyruvate metabolism | 20 | 2.94 | 1 | 9.60E-01 |
| Arachidonic acid metabolism | 37 | 5.43 | 2 | 9.81E-01 |
| Tyrosine metabolism | 38 | 5.58 | 2 | 9.84E-01 |
| Steroid biosynthesis | 31 | 4.55 | 1 | 9.93E-01 |
| Valine, leucine and isoleucine degradation | 36 | 5.29 | 1 | 9.97E-01 |

Table S3 Raw metabolomic data.

| Metabolite | Control-1 | Control-2 | Control-3 | Control-4 | Control-5 | Control-6 | Control-7 | Control-8 | Control-9 | Celastrol-1 | Celastrol-2 | Celastrol-3 | Celastrol-4 | Celastrol-5 | Celastrol-6 | Celastrol-7 | Celastrol-8 | Celastrol-9 |
| --- | --- | --- | --- | --- | --- | --- | --- | --- | --- | --- | --- | --- | --- | --- | --- | --- | --- | --- |
| 17-Hydroxyprogesterone | 3.46E-01 | 5.87E-01 | 8.80E-01 | 2.52E+00 | 6.11E-01 | 2.25E-01 | 1.09E+00 | 1.41E-01 | 9.56E-02 | 1.25E-01 | 8.97E-01 | 1.22E+00 | 1.57E+00 | 6.39E-02 | 2.54E+00 | 7.54E-01 | 1.66E+00 | 2.67E+00 |
| 2-Methoxyestrone | 9.99E-01 | 2.51E+00 | 5.63E-01 | 1.33E+00 | 5.63E-01 | 1.99E+00 | 5.63E-01 | 5.63E-01 | 5.63E-01 | 1.72E+00 | 1.13E+00 | 5.63E-01 | 1.83E+00 | 5.63E-01 | 8.53E-01 | 5.63E-01 | 5.63E-01 | 5.63E-01 |
| 3-Indoleacetonitrile | 6.90E-01 | 1.48E+00 | 6.90E-01 | 6.90E-01 | 6.90E-01 | 6.90E-01 | 6.90E-01 | 1.03E+00 | 1.60E+00 | 7.17E-01 | 6.90E-01 | 1.73E+00 | 2.05E+00 | 1.02E+00 | 6.90E-01 | 1.47E+00 | 6.90E-01 | 6.90E-01 |
| 3-Indolepropionic acid | 1.83E-01 | 1.83E-01 | 1.83E-01 | 3.23E-01 | 1.83E-01 | 6.93E-01 | 1.83E-01 | 1.83E-01 | 1.83E-01 | 1.83E-01 | 1.93E-01 | 1.83E-01 | 7.66E-01 | 1.83E-01 | 8.26E-01 | 4.19E-01 | 3.50E+00 | 9.45E+00 |
| 3-Methyladenine | 7.88E-01 | 4.44E-01 | 8.20E-01 | 2.76E+00 | 1.08E+00 | 2.61E+00 | 2.51E+00 | 1.03E+00 | 8.68E-01 | 7.20E-01 | 4.08E-01 | 6.95E-02 | 9.94E-01 | 3.09E-02 | 5.40E-01 | 1.65E-01 | 5.18E-01 | 1.64E+00 |
| 3-Methylhistidine | 6.18E-01 | 9.01E-01 | 1.26E+00 | 1.62E+00 | 3.40E-01 | 1.22E+00 | 2.07E+00 | 1.38E+00 | 1.92E-01 | 7.70E-01 | 5.58E-01 | 7.19E-01 | 1.21E+00 | 8.74E-01 | 1.07E+00 | 1.20E+00 | 8.17E-01 | 1.19E+00 |
| 3-Methylxanthine | 4.93E-01 | 8.95E-01 | 1.59E+00 | 4.82E-01 | 1.10E+00 | 4.82E-01 | 1.62E+00 | 1.12E+00 | 8.81E-01 | 6.60E-01 | 9.84E-01 | 1.28E+00 | 4.82E-01 | 2.38E+00 | 9.43E-01 | 1.42E+00 | 4.82E-01 | 7.10E-01 |
| 3-Pyridylacetic acid | 6.90E-01 | 5.71E-01 | 7.88E-01 | 9.42E-01 | 4.25E-01 | 7.51E-01 | 9.80E-01 | 8.95E-01 | 6.55E-01 | 1.72E+00 | 1.13E+00 | 2.64E-01 | 1.66E+00 | 2.01E-01 | 7.59E-01 | 6.51E-01 | 1.77E+00 | 3.15E+00 |
| 4-Hydroxyproline | 1.49E+00 | 1.61E+00 | 3.94E+00 | 1.19E+00 | 8.97E-01 | 1.18E+00 | 3.32E+00 | 2.86E+00 | 1.97E-01 | 1.05E-01 | 1.05E-01 | 1.09E-01 | 1.05E-01 | 1.05E-01 | 3.68E-01 | 1.71E-01 | 1.41E-01 | 1.05E-01 |
| 4-Methylcatechol | 1.69E+00 | 8.98E-01 | 6.80E-01 | 6.80E-01 | 7.26E-01 | 6.80E-01 | 6.80E-01 | 6.80E-01 | 6.80E-01 | 6.80E-01 | 1.31E+00 | 2.24E+00 | 1.09E+00 | 1.15E+00 | 1.30E+00 | 7.66E-01 | 1.39E+00 | 6.80E-01 |
| 5-Hydroxylysine | 1.05E+00 | 7.20E-01 | 1.34E+00 | 2.21E+00 | 8.98E-01 | 2.12E+00 | 1.82E+00 | 1.34E+00 | 7.84E-01 | 7.18E-01 | 4.37E-01 | 2.80E-01 | 9.42E-01 | 2.91E-01 | 7.95E-01 | 5.23E-01 | 5.58E-01 | 1.17E+00 |
| 5-Methoxytryptamine | 7.19E-01 | 5.32E-01 | 1.91E+00 | 4.08E+00 | 5.89E-01 | 2.22E+00 | 1.67E+00 | 5.32E-01 | 5.32E-01 | 6.77E-01 | 5.32E-01 | 5.32E-01 | 5.32E-01 | 5.32E-01 | 5.32E-01 | 5.32E-01 | 5.32E-01 | 8.20E-01 |
| 5'-Methylthioadenosine | 3.62E-01 | 6.01E-01 | 1.14E+00 | 8.52E-01 | 6.20E-01 | 1.76E+00 | 2.37E+00 | 7.11E+00 | 9.09E-01 | 4.06E-01 | 1.95E-01 | 3.51E-02 | 3.76E-01 | 3.42E-02 | 1.60E-01 | 1.07E-01 | 1.60E-01 | 7.99E-01 |
| 7b-Hydroxycholesterol | 6.51E-01 | 4.29E-01 | 9.07E-02 | 2.59E-01 | 9.07E-02 | 5.68E+00 | 4.46E-01 | 1.80E-01 | 4.23E-01 | 5.06E-01 | 2.48E+00 | 1.47E-01 | 3.80E-01 | 1.90E-01 | 3.30E-01 | 9.09E-01 | 5.97E-01 | 4.21E+00 |
| Acetylcysteine | 5.07E-01 | 6.62E-01 | 1.31E+00 | 2.99E+00 | 3.86E-01 | 1.28E+00 | 4.64E+00 | 1.22E+00 | 8.43E-02 | 8.43E-02 | 4.94E-01 | 8.43E-02 | 6.24E-01 | 1.22E-01 | 8.43E-02 | 7.60E-01 | 1.75E+00 | 9.29E-01 |
| Acetylglycine | 2.27E-01 | 3.22E-01 | 1.98E-01 | 1.98E-01 | 7.01E-01 | 1.23E+00 | 5.77E-01 | 4.00E-01 | 4.28E-01 | 7.59E-01 | 9.24E-01 | 6.48E-01 | 5.10E-01 | 1.40E+00 | 4.74E+00 | 1.11E+00 | 1.82E+00 | 1.81E+00 |
| Adenine | 7.96E-01 | 1.21E+00 | 1.96E+00 | 2.25E+00 | 4.89E-01 | 1.90E+00 | 2.62E+00 | 1.85E+00 | 5.24E-01 | 4.89E-01 | 4.89E-01 | 4.89E-01 | 4.89E-01 | 4.89E-01 | 4.89E-01 | 4.89E-01 | 4.89E-01 | 4.89E-01 |
| Adenosine | 9.24E-02 | 9.24E-02 | 9.24E-02 | 9.24E-02 | 1.26E-01 | 9.24E-02 | 9.24E-02 | 9.24E-02 | 9.24E-02 | 8.07E-01 | 1.93E-01 | 9.24E-02 | 2.15E-01 | 1.88E-01 | 2.11E-01 | 4.40E-01 | 2.89E+00 | 1.21E+01 |
| Allantoin | 5.83E-01 | 6.12E-01 | 3.43E-01 | 2.28E-01 | 9.15E-01 | 4.62E-01 | 2.49E-01 | 5.72E-01 | 7.95E-01 | 9.21E-01 | 1.60E+00 | 1.35E+00 | 1.25E+00 | 1.70E+00 | 1.83E+00 | 2.05E+00 | 1.71E+00 | 8.29E-01 |
| AMP | 5.61E-02 | 2.35E+00 | 2.40E+00 | 3.68E-01 | 1.70E-02 | 3.32E-01 | 5.82E+00 | 5.80E+00 | 1.07E-01 | 5.15E-03 | 3.09E-03 | 2.34E-03 | 2.34E-03 | 2.79E-03 | 2.34E-03 | 3.53E-03 | 2.00E-01 | 5.26E-01 |
| Arachidonic acid | 7.52E-03 | 7.36E-02 | 1.34E+00 | 7.52E-03 | 2.34E-02 | 1.59E+01 | 7.52E-03 | 5.40E-02 | 7.52E-03 | 2.73E-02 | 5.66E-02 | 1.45E-01 | 2.51E-01 | 7.52E-03 | 7.52E-03 | 7.52E-03 | 1.99E-02 | 2.04E-02 |
| Argininosuccinic acid | 4.56E-01 | 1.39E+00 | 1.51E+00 | 1.65E+00 | 2.05E+00 | 1.01E+00 | 6.81E-01 | 7.76E-01 | 1.44E+00 | 4.85E-01 | 5.74E-01 | 1.10E+00 | 1.18E+00 | 5.73E-01 | 9.84E-01 | 4.41E-01 | 9.17E-01 | 7.80E-01 |
| Ascorbic acid | 7.16E-01 | 1.91E+00 | 2.29E+00 | 2.53E+00 | 5.79E-02 | 7.10E-01 | 5.56E+00 | 2.47E+00 | 3.18E-02 | 2.52E-01 | 1.58E-01 | 4.74E-02 | 2.30E-01 | 5.80E-02 | 3.89E-01 | 8.84E-02 | 1.37E-01 | 3.69E-01 |
| Beta-Alanine | 2.06E-01 | 1.31E-01 | 1.31E-01 | 4.24E+00 | 4.31E-01 | 4.31E+00 | 2.74E+00 | 1.44E-01 | 2.25E-01 | 8.91E-01 | 5.05E-01 | 1.31E-01 | 1.04E+00 | 5.50E-01 | 4.91E-01 | 5.95E-01 | 1.31E-01 | 1.11E+00 |
| Bilirubin | 4.54E-01 | 1.01E+00 | 2.46E+00 | 9.89E+00 | 1.45E-01 | 2.49E-01 | 1.45E-01 | 5.78E-01 | 1.45E-01 | 2.33E-01 | 3.33E-01 | 2.32E-01 | 2.54E-01 | 3.28E-01 | 6.11E-01 | 1.45E-01 | 1.45E-01 | 6.43E-01 |
| Biotin | 3.34E-01 | 3.19E-01 | 3.71E-01 | 3.29E+00 | 3.69E-01 | 2.47E+00 | 1.65E+00 | 2.23E+00 | 2.77E-01 | 8.23E-01 | 6.88E-01 | 2.10E-01 | 1.14E+00 | 2.10E-01 | 7.05E-01 | 2.10E-01 | 8.19E-01 | 1.88E+00 |
| Carnosine | 7.80E-01 | 1.39E+00 | 2.00E+00 | 1.34E+00 | 3.34E-01 | 5.73E-01 | 7.14E-01 | 5.01E+00 | 6.62E-01 | 7.17E-01 | 5.62E-01 | 3.70E-02 | 1.34E+00 | 3.70E-02 | 1.06E+00 | 6.54E-02 | 5.32E-01 | 8.60E-01 |
| Cholesterol | 1.18E+00 | 1.39E+00 | 9.20E-01 | 9.64E-01 | 1.60E-01 | 7.75E-01 | 1.48E+00 | 9.02E-01 | 8.54E-01 | 1.07E+00 | 6.66E-01 | 9.65E-01 | 1.17E+00 | 9.92E-01 | 1.21E+00 | 3.62E-01 | 1.22E+00 | 1.71E+00 |
| Cholic acid | 6.73E-02 | 6.73E-02 | 6.73E-02 | 2.28E-01 | 3.34E-01 | 1.06E+00 | 2.70E-01 | 6.73E-02 | 6.73E-02 | 3.05E-01 | 1.95E+00 | 3.95E-01 | 3.99E+00 | 1.32E+00 | 2.08E+00 | 1.44E+00 | 7.22E-01 | 3.57E+00 |
| cis-Aconitic acid | 9.40E-01 | 3.87E-01 | 2.17E-01 | 9.00E-01 | 1.13E+00 | 3.86E+00 | 7.49E-01 | 5.85E-01 | 1.03E+00 | 1.17E+00 | 5.29E-01 | 2.35E-01 | 8.88E-01 | 3.54E-01 | 1.44E+00 | 6.39E-01 | 8.62E-01 | 2.11E+00 |
| Citric acid | 1.14E+00 | 3.85E-01 | 3.97E-01 | 8.62E-01 | 1.10E+00 | 1.69E+00 | 1.17E+00 | 5.49E-01 | 1.03E+00 | 1.34E+00 | 5.90E-01 | 2.82E-01 | 9.79E-01 | 4.19E-01 | 1.71E+00 | 7.02E-01 | 1.05E+00 | 2.62E+00 |
| Cortisone | 5.20E-02 | 5.20E-02 | 6.26E-02 | 4.32E-01 | 1.31E-01 | 8.48E-01 | 2.56E-01 | 5.20E-02 | 5.20E-02 | 1.97E+00 | 2.42E+00 | 2.72E-01 | 5.25E+00 | 5.69E-01 | 1.74E+00 | 4.56E-01 | 8.32E-01 | 2.55E+00 |
| Creatinine | 5.36E-01 | 5.20E-01 | 6.69E-01 | 1.33E+00 | 4.49E-01 | 1.49E+00 | 1.03E+00 | 2.28E+00 | 4.75E-01 | 1.33E+00 | 7.55E-01 | 2.36E-01 | 1.40E+00 | 2.36E-01 | 1.29E+00 | 5.06E-01 | 1.06E+00 | 2.41E+00 |
| Cyclic AMP | 1.39E+00 | 7.65E-01 | 1.19E+00 | 2.19E+00 | 4.80E-02 | 8.44E-01 | 3.93E+00 | 5.87E+00 | 8.92E-02 | 7.57E-03 | 7.57E-03 | 7.57E-03 | 1.79E-02 | 7.57E-03 | 3.57E-02 | 1.29E-02 | 5.98E-02 | 1.53E+00 |
| Cytidine | 4.29E-02 | 7.29E-02 | 1.17E-01 | 5.33E-01 | 2.93E-01 | 1.17E+00 | 5.48E-01 | 7.13E+00 | 8.80E-01 | 5.23E-01 | 1.45E-01 | 4.97E-02 | 4.97E-01 | 1.45E-01 | 1.20E+00 | 9.30E-02 | 1.62E+00 | 2.94E+00 |
| Deoxycytidine | 4.67E-01 | 7.74E-01 | 9.05E-01 | 1.24E+00 | 3.86E-01 | 1.44E+00 | 1.70E+00 | 1.44E+00 | 3.87E-01 | 3.27E-01 | 5.04E-02 | 2.82E-02 | 4.46E-01 | 3.17E-01 | 1.27E+00 | 1.92E-01 | 2.24E+00 | 4.40E+00 |
| Deoxyinosine | 1.32E+00 | 5.73E-01 | 1.09E+00 | 4.59E+00 | 1.02E+00 | 4.27E+00 | 2.86E+00 | 1.50E+00 | 5.02E-01 | 2.20E-02 | 4.70E-02 | 1.86E-02 | 9.60E-02 | 1.86E-02 | 1.86E-02 | 1.86E-02 | 1.86E-02 | 1.86E-02 |
| D-Glucaric acid | 4.82E-01 | 4.18E-01 | 7.23E-01 | 4.17E+00 | 8.50E-01 | 3.77E+00 | 3.83E+00 | 1.84E+00 | 4.00E-01 | 1.86E-01 | 1.65E-01 | 1.65E-01 | 1.65E-01 | 1.65E-01 | 1.65E-01 | 1.65E-01 | 1.65E-01 | 1.65E-01 |
| D-Glucuronic acid | 4.64E-01 | 3.65E-01 | 3.65E-01 | 3.28E+00 | 1.06E+00 | 4.00E+00 | 1.25E+00 | 3.73E-01 | 1.24E+00 | 3.65E-01 | 3.65E-01 | 3.65E-01 | 9.26E-01 | 3.65E-01 | 8.76E-01 | 3.65E-01 | 8.96E-01 | 1.09E+00 |
| D-Glucurono-6,3-lactone | 8.50E-02 | 8.50E-02 | 8.50E-02 | 8.50E-02 | 8.50E-02 | 8.50E-02 | 8.50E-02 | 8.50E-02 | 8.50E-02 | 4.18E+00 | 1.72E+00 | 9.42E-02 | 4.23E+00 | 6.39E-01 | 1.76E+00 | 6.13E-01 | 1.10E+00 | 2.90E+00 |
| Dihydroxyacetone phosphate | 1.22E+00 | 8.65E-01 | 9.51E-01 | 6.09E-01 | 1.55E+00 | 5.46E-01 | 7.51E-01 | 1.20E+00 | 1.44E+00 | 1.17E+00 | 1.04E+00 | 7.48E-01 | 7.80E-01 | 6.82E-01 | 1.36E+00 | 1.08E+00 | 1.21E+00 | 7.99E-01 |
| Dodecanedioic acid | 7.44E-01 | 1.64E-01 | 8.69E-01 | 1.51E+00 | 3.79E-01 | 2.16E+00 | 1.11E+00 | 1.24E+00 | 5.74E-01 | 1.15E+00 | 7.01E-01 | 4.15E-01 | 1.78E+00 | 4.99E-01 | 1.06E+00 | 5.94E-01 | 8.37E-01 | 2.21E+00 |
| Fumaric acid | 5.61E-01 | 1.31E+00 | 1.31E+00 | 1.13E+00 | 8.66E-01 | 6.66E-01 | 5.63E+00 | 3.01E+00 | 3.33E-01 | 4.12E-01 | 2.12E-01 | 4.31E-02 | 5.91E-01 | 4.47E-02 | 2.98E-01 | 4.31E-02 | 3.00E-01 | 1.24E+00 |
| Gluconolactone | 1.57E+00 | 1.66E+00 | 8.72E-01 | 2.98E+00 | 1.43E+00 | 2.41E+00 | 1.94E+00 | 2.81E-01 | 9.95E-01 | 1.02E+00 | 2.10E-01 | 1.93E-01 | 1.04E+00 | 7.00E-02 | 1.95E-01 | 2.18E-01 | 5.00E-01 | 4.19E-01 |
| Glucosamine 6-phosphate | 1.33E+00 | 5.98E-01 | 1.42E+00 | 3.46E+00 | 1.02E+00 | 5.49E+00 | 2.82E+00 | 1.42E+00 | 3.74E-01 | 6.82E-03 | 6.82E-03 | 6.82E-03 | 6.82E-03 | 6.82E-03 | 7.17E-03 | 6.82E-03 | 6.82E-03 | 1.35E-02 |
| Glucose 6-phosphate | 2.24E+00 | 2.45E+00 | 2.95E+00 | 2.04E+00 | 1.23E+00 | 1.42E+00 | 1.76E+00 | 2.60E+00 | 1.11E+00 | 6.07E-02 | 2.59E-02 | 1.26E-02 | 4.54E-02 | 3.07E-02 | 1.04E-02 | 6.67E-03 | 1.16E-02 | 7.39E-03 |
| Glutamic acid | 1.34E+00 | 2.31E+00 | 2.69E+00 | 2.02E+00 | 9.01E-01 | 1.50E+00 | 2.73E+00 | 3.80E+00 | 5.14E-01 | 4.39E-02 | 2.12E-02 | 1.37E-02 | 2.75E-02 | 1.21E-02 | 2.78E-02 | 1.19E-02 | 1.84E-02 | 2.23E-02 |
| Glyceraldehyde | 3.02E-01 | 1.63E-01 | 9.09E-02 | 2.48E-01 | 1.77E-01 | 2.14E-01 | 1.21E-01 | 1.20E-01 | 1.61E-01 | 1.85E+00 | 1.76E+00 | 7.38E-01 | 1.74E+00 | 1.03E+00 | 3.38E+00 | 1.89E+00 | 2.17E+00 | 1.84E+00 |
| Glycerophosphocholine | 3.28E-01 | 1.30E+00 | 2.46E+00 | 4.18E-01 | 4.22E-01 | 3.19E-01 | 1.05E+00 | 7.79E+00 | 3.83E-01 | 4.83E-01 | 4.33E-01 | 3.56E-01 | 3.49E-01 | 4.12E-01 | 8.23E-01 | 3.49E-01 | 2.02E-01 | 1.26E-01 |
| Glycine | 1.88E+00 | 1.05E+00 | 5.48E-01 | 1.25E+00 | 1.23E+00 | 1.57E+00 | 6.60E-01 | 7.74E-01 | 1.40E+00 | 9.02E-01 | 4.37E-01 | 3.25E-01 | 9.43E-01 | 5.69E-01 | 1.77E+00 | 5.00E-01 | 1.02E+00 | 1.19E+00 |
| Glycolic acid | 1.78E-01 | 6.81E-02 | 6.81E-02 | 6.81E-02 | 1.53E-01 | 3.82E-01 | 6.81E-02 | 6.81E-02 | 1.89E-01 | 5.16E+00 | 1.65E+00 | 1.05E-01 | 4.14E+00 | 6.81E-02 | 1.52E+00 | 4.31E-01 | 7.80E-01 | 2.90E+00 |
| Guanine | 5.18E-01 | 1.17E+00 | 1.49E+00 | 1.79E+00 | 2.54E-01 | 1.48E+00 | 3.52E+00 | 1.41E+00 | 4.11E-01 | 4.37E-03 | 5.32E+00 | 4.37E-03 | 4.37E-03 | 2.33E-01 | 4.35E-02 | 1.68E-02 | 1.17E-01 | 2.10E-01 |
| Hexadecanedioic acid | 2.17E-01 | 2.17E-01 | 2.17E-01 | 2.17E-01 | 2.17E-01 | 9.84E-01 | 2.17E-01 | 2.17E-01 | 2.17E-01 | 2.17E+00 | 8.70E-01 | 5.22E-01 | 2.41E+00 | 7.87E-01 | 2.39E+00 | 5.10E-01 | 1.41E+00 | 4.22E+00 |
| Hippuric acid | 2.86E-01 | 2.86E-01 | 3.16E-01 | 5.05E-01 | 2.86E-01 | 7.33E-01 | 4.01E-01 | 4.93E-01 | 2.86E-01 | 9.29E-01 | 1.14E+00 | 2.86E-01 | 2.84E+00 | 2.86E-01 | 7.14E-01 | 5.72E-01 | 1.98E+00 | 5.66E+00 |
| Histamine | 4.97E-01 | 7.10E-01 | 1.06E+00 | 3.24E+00 | 1.80E-01 | 5.59E-01 | 8.48E-01 | 7.99E-01 | 9.71E-02 | 3.94E+00 | 5.64E-01 | 9.71E-02 | 2.97E+00 | 9.71E-02 | 3.13E-01 | 9.71E-02 | 9.71E-02 | 1.83E+00 |
| Homocysteine | 8.65E-01 | 1.02E+00 | 1.46E+00 | 2.25E+00 | 1.01E+00 | 2.26E+00 | 2.64E+00 | 1.37E+00 | 4.24E-01 | 7.09E-01 | 4.41E-01 | 1.90E-01 | 6.37E-01 | 2.57E-01 | 1.10E+00 | 3.57E-01 | 3.47E-01 | 6.77E-01 |
| Hypotaurine | 1.41E+00 | 8.19E-01 | 1.17E+00 | 9.02E-01 | 1.68E+00 | 1.54E+00 | 6.60E-01 | 1.91E+00 | 1.37E+00 | 8.02E-01 | 6.08E-01 | 3.88E-01 | 6.03E-01 | 3.74E-01 | 1.05E+00 | 4.86E-01 | 1.01E+00 | 1.22E+00 |
| Indoleacetic acid | 2.01E-01 | 2.01E-01 | 2.01E-01 | 2.01E-01 | 2.01E-01 | 2.01E-01 | 2.01E-01 | 2.01E-01 | 2.01E-01 | 1.94E+00 | 1.05E+00 | 2.31E-01 | 2.39E+00 | 2.01E-01 | 1.06E+00 | 5.49E-01 | 2.15E+00 | 6.62E+00 |
| Inosine | 4.33E-01 | 9.02E-01 | 1.68E+00 | 2.93E+00 | 1.14E-01 | 2.18E+00 | 3.76E+00 | 2.33E+00 | 4.04E-01 | 1.97E-01 | 6.05E-01 | 1.54E-02 | 1.08E-01 | 2.06E-01 | 5.60E-01 | 1.35E-01 | 2.99E-01 | 1.13E+00 |
| Inosinic acid | 3.50E-03 | 3.20E+00 | 4.06E+00 | 5.70E-01 | 1.70E-03 | 5.13E-01 | 5.66E+00 | 3.97E+00 | 1.24E-02 | 5.22E-04 | 7.26E-05 | 2.75E-05 | 1.04E-04 | 5.07E-04 | 1.29E-04 | 2.33E-04 | 2.58E-03 | 3.26E-03 |
| Itaconic acid | 9.23E-01 | 4.92E-01 | 8.78E-01 | 2.14E+00 | 1.18E+00 | 2.53E+00 | 2.10E+00 | 9.34E-01 | 8.00E-01 | 1.13E+00 | 4.45E-01 | 2.63E-01 | 8.65E-01 | 1.65E-01 | 9.16E-01 | 5.09E-01 | 5.31E-01 | 1.20E+00 |
| L-Acetylcarnitine | 1.21E+00 | 9.39E-01 | 1.31E+00 | 2.33E+00 | 8.38E-01 | 2.08E+00 | 2.83E+00 | 1.82E+00 | 6.08E-01 | 6.54E-01 | 3.77E-01 | 1.09E-01 | 4.83E-01 | 1.26E-01 | 5.58E-01 | 1.98E-01 | 4.19E-01 | 1.12E+00 |
| L-Aspartyl-L-phenylalanine | 2.51E-01 | 1.41E+00 | 2.33E+00 | 3.34E+00 | 3.62E-01 | 1.13E+00 | 3.95E+00 | 4.30E+00 | 2.34E-01 | 1.91E-01 | 8.44E-02 | 8.31E-03 | 1.59E-01 | 2.22E-03 | 3.48E-02 | 1.75E-02 | 4.76E-02 | 1.44E-01 |
| L-Carnitine | 9.52E-01 | 1.07E+00 | 1.54E+00 | 2.51E+00 | 5.26E-01 | 2.13E+00 | 2.97E+00 | 1.92E+00 | 3.39E-01 | 8.46E-01 | 5.17E-01 | 1.01E-01 | 6.82E-01 | 8.33E-02 | 2.67E-01 | 2.39E-01 | 4.29E-01 | 8.74E-01 |
| L-Cysteine | 5.48E-01 | 2.08E+00 | 5.20E+00 | 2.32E+00 | 1.55E-01 | 6.65E-01 | 4.50E+00 | 1.76E+00 | 7.70E-02 | 7.70E-02 | 7.70E-02 | 7.70E-02 | 7.70E-02 | 7.70E-02 | 7.70E-02 | 7.70E-02 | 7.70E-02 | 7.70E-02 |
| L-Cystine | 2.69E+00 | 1.06E-01 | 3.18E-01 | 3.24E+00 | 1.79E+00 | 6.98E+00 | 2.06E-01 | 1.58E-01 | 1.05E+00 | 4.76E-02 | 5.73E-02 | 4.03E-02 | 2.48E-01 | 1.17E-02 | 9.10E-02 | 3.33E-02 | 7.03E-01 | 2.35E-01 |
| Levulinic acid | 1.35E+00 | 1.42E+00 | 7.94E-01 | 8.60E-01 | 1.05E+00 | 1.70E+00 | 1.21E+00 | 4.07E-01 | 1.50E+00 | 3.59E-01 | 1.20E+00 | 8.04E-01 | 1.77E+00 | 1.09E+00 | 9.88E-01 | 5.68E-01 | 5.61E-01 | 3.59E-01 |
| L-Histidine | 1.09E+00 | 8.02E-01 | 6.37E-01 | 1.61E+00 | 6.47E-01 | 2.09E+00 | 8.15E-01 | 4.51E-01 | 4.78E-01 | 1.71E+00 | 9.18E-01 | 3.43E-01 | 7.91E-01 | 3.88E-01 | 1.47E+00 | 7.60E-01 | 1.42E+00 | 1.57E+00 |
| Linoleic acid | 1.02E-01 | 5.59E-01 | 2.35E+00 | 3.46E-01 | 1.02E-01 | 8.16E+00 | 7.91E-01 | 8.30E-01 | 3.51E-01 | 1.99E-01 | 2.70E-01 | 3.19E-01 | 7.46E-01 | 9.35E-01 | 1.21E+00 | 1.02E-01 | 3.14E-01 | 3.13E-01 |
| L-Leucine | 6.46E-01 | 5.42E-01 | 8.95E-01 | 2.67E+00 | 6.46E-01 | 2.25E+00 | 2.31E+00 | 1.15E+00 | 4.23E-01 | 1.05E+00 | 5.71E-01 | 1.01E-01 | 1.04E+00 | 9.36E-02 | 6.24E-01 | 2.51E-01 | 5.78E-01 | 2.16E+00 |
| L-Lysine | 1.00E+00 | 8.62E-01 | 1.68E+00 | 1.84E+00 | 1.12E+00 | 1.58E+00 | 1.50E+00 | 1.17E+00 | 7.84E-01 | 6.37E-01 | 4.17E-01 | 3.01E-01 | 7.37E-01 | 3.69E-01 | 1.15E+00 | 6.03E-01 | 7.73E-01 | 1.46E+00 |
| L-Malic acid | 9.35E-01 | 3.61E-01 | 2.92E-01 | 3.45E+00 | 1.08E+00 | 3.28E+00 | 1.64E+00 | 4.53E-01 | 9.59E-01 | 1.07E+00 | 3.77E-01 | 1.04E-01 | 7.09E-01 | 1.27E-01 | 9.39E-01 | 4.63E-01 | 5.50E-01 | 1.21E+00 |
| L-Palmitoylcarnitine | 6.56E-02 | 3.91E+00 | 7.74E-01 | 6.55E-01 | 5.94E-02 | 1.29E+00 | 1.61E+00 | 3.20E+00 | 5.13E-01 | 3.09E-02 | 2.78E-01 | 3.09E-02 | 2.99E-01 | 5.97E-02 | 1.57E+00 | 3.09E-02 | 1.08E-01 | 3.51E+00 |
| L-Phenylalanine | 1.31E-01 | 2.83E-01 | 7.38E-01 | 2.68E+00 | 2.59E-01 | 2.93E+00 | 2.20E+00 | 6.70E-01 | 1.15E-01 | 1.39E+00 | 1.15E-01 | 1.15E-01 | 1.37E+00 | 1.15E-01 | 8.56E-01 | 1.72E-01 | 4.00E-01 | 3.46E+00 |
| L-Proline | 1.46E+00 | 1.16E+00 | 1.91E+00 | 2.94E+00 | 8.68E-01 | 2.32E+00 | 3.31E+00 | 2.38E+00 | 5.86E-01 | 1.09E-01 | 5.25E-02 | 3.26E-02 | 1.21E-01 | 3.79E-02 | 2.10E-01 | 1.03E-01 | 1.42E-01 | 2.50E-01 |
| L-Serine | 8.17E-01 | 3.23E-01 | 2.01E-01 | 1.05E+00 | 8.73E-01 | 9.69E-01 | 2.01E-01 | 1.20E-01 | 5.27E-01 | 1.82E+00 | 1.38E+00 | 8.61E-01 | 1.62E+00 | 7.98E-01 | 1.90E+00 | 9.60E-01 | 1.81E+00 | 1.77E+00 |
| L-Tryptophan | 3.83E-01 | 2.88E-01 | 4.62E-01 | 1.59E+00 | 3.75E-01 | 1.32E+00 | 1.24E+00 | 4.73E-01 | 2.03E-01 | 1.98E+00 | 1.06E+00 | 2.76E-01 | 2.07E+00 | 3.33E-01 | 1.73E+00 | 6.47E-01 | 9.12E-01 | 2.66E+00 |
| Maleic acid | 6.21E-01 | 5.23E-01 | 8.09E-01 | 2.31E+00 | 1.33E+00 | 1.01E+00 | 9.16E-01 | 1.04E+00 | 1.32E+00 | 7.53E-01 | 1.11E+00 | 2.93E-01 | 8.64E-01 | 1.09E-01 | 1.15E+00 | 1.13E+00 | 6.32E-01 | 2.08E+00 |
| Melatonin | 3.80E-01 | 3.80E-01 | 3.80E-01 | 3.80E-01 | 3.80E-01 | 1.32E+00 | 1.88E+00 | 3.80E-01 | 3.80E-01 | 3.80E-01 | 1.11E+00 | 3.80E-01 | 8.04E-01 | 1.00E+00 | 3.83E+00 | 3.80E+00 | 3.80E-01 | 4.60E-01 |
| N-Acetylglutamic acid | 7.14E-01 | 6.63E-01 | 9.32E-01 | 2.70E+00 | 4.92E-01 | 2.42E+00 | 3.07E+00 | 2.88E+00 | 3.73E-01 | 2.31E-01 | 1.38E-01 | 1.38E-01 | 4.31E-01 | 1.38E-01 | 3.81E-01 | 2.11E-01 | 3.79E-01 | 1.71E+00 |
| N-Acetylglutamine | 2.90E-01 | 4.74E-01 | 4.62E-01 | 1.06E+00 | 2.92E-01 | 1.02E+00 | 2.03E+00 | 1.65E+00 | 3.11E-01 | 1.33E+00 | 6.51E-01 | 1.36E-01 | 1.39E+00 | 1.93E-01 | 1.25E+00 | 4.23E-01 | 1.25E+00 | 3.79E+00 |
| N-Acetylleucine | 1.65E-01 | 1.65E-01 | 1.65E-01 | 1.95E-01 | 2.67E-01 | 1.31E+00 | 3.40E-01 | 3.48E-01 | 1.86E-01 | 1.39E+00 | 4.87E-01 | 1.65E-01 | 1.59E+00 | 5.40E-01 | 3.19E+00 | 3.79E-01 | 1.25E+00 | 5.87E+00 |
| N-Acetyl-L-methionine | 9.63E-01 | 9.28E-01 | 1.76E+00 | 2.20E+00 | 6.33E-01 | 3.18E+00 | 3.86E+00 | 2.07E+00 | 2.92E-01 | 3.58E-01 | 1.16E-01 | 2.05E-02 | 3.21E-01 | 2.67E-02 | 1.69E-01 | 1.00E-01 | 2.92E-01 | 7.08E-01 |
| N-Acetyl-L-phenylalanine | 2.60E-02 | 2.88E-02 | 3.12E-02 | 4.16E-02 | 2.17E-02 | 4.62E-02 | 8.11E-02 | 4.84E-02 | 2.17E-02 | 3.55E+00 | 9.34E-01 | 1.62E-01 | 2.56E+00 | 1.60E-01 | 1.06E+00 | 7.45E-01 | 1.68E+00 | 6.79E+00 |
| N-Acetyl-L-tyrosine | 7.48E-02 | 1.65E-02 | 1.97E-02 | 5.90E-02 | 3.78E-02 | 1.65E-01 | 1.65E-02 | 2.15E-01 | 1.65E-02 | 2.52E-02 | 1.65E-02 | 1.65E-02 | 1.65E-02 | 1.65E-02 | 1.65E-02 | 2.80E-02 | 1.65E-02 | 1.72E+01 |
| N-Acetylneuraminic acid | 2.25E+00 | 1.42E+00 | 1.17E+00 | 3.02E+00 | 1.28E+00 | 3.02E+00 | 1.92E+00 | 1.23E+00 | 9.90E-01 | 3.98E-01 | 4.05E-01 | 4.47E-02 | 3.79E-01 | 4.57E-02 | 1.25E-01 | 6.97E-02 | 1.21E-01 | 1.19E-01 |
| N-Acetylvaline | 3.50E-01 | 2.30E-01 | 2.30E-01 | 4.17E-01 | 2.30E-01 | 2.30E-01 | 2.30E-01 | 7.68E-01 | 4.59E-01 | 1.30E+00 | 6.40E-01 | 2.30E-01 | 1.95E+00 | 2.30E-01 | 1.87E+00 | 2.30E-01 | 1.99E+00 | 6.41E+00 |
| N-Alpha-acetyllysine | 7.20E-01 | 9.25E-01 | 1.31E+00 | 1.70E+00 | 9.35E-01 | 1.57E+00 | 1.86E+00 | 1.50E+00 | 4.76E-01 | 1.06E+00 | 7.28E-01 | 1.62E-01 | 1.40E+00 | 1.58E-01 | 8.92E-01 | 3.83E-01 | 6.39E-01 | 1.57E+00 |
| N-Formyl-L-methionine | 5.00E-01 | 1.19E+00 | 1.23E+00 | 2.32E+00 | 4.16E-01 | 1.98E+00 | 3.05E+00 | 2.97E+00 | 2.17E-01 | 1.33E+00 | 4.93E-01 | 5.33E-02 | 1.43E+00 | 5.33E-02 | 4.86E-01 | 1.82E-01 | 5.33E-02 | 5.33E-02 |
| Niacinamide | 9.43E-01 | 8.77E-01 | 1.56E+00 | 3.50E+00 | 5.90E-01 | 2.72E+00 | 4.03E+00 | 2.10E+00 | 4.80E-01 | 2.01E-01 | 7.27E-02 | 1.71E-02 | 1.55E-01 | 9.12E-03 | 6.37E-02 | 4.74E-02 | 1.74E-01 | 4.66E-01 |
| N-Oleoylethanolamine | 2.80E-01 | 1.07E+00 | 7.72E-01 | 4.58E-01 | 2.21E-01 | 1.28E+01 | 3.01E-01 | 6.44E-01 | 4.62E-01 | 2.17E-02 | 2.17E-02 | 2.17E-02 | 8.50E-02 | 2.17E-02 | 7.59E-01 | 2.17E-02 | 2.17E-02 | 2.17E-02 |
| Norvaline | 6.71E-01 | 6.05E-01 | 9.28E-01 | 1.57E+00 | 5.85E-01 | 1.04E+00 | 1.35E+00 | 1.30E+00 | 5.02E-01 | 1.28E+00 | 8.40E-01 | 2.02E-01 | 1.58E+00 | 1.70E-01 | 7.95E-01 | 3.67E-01 | 9.48E-01 | 3.27E+00 |
| Octadecanamide | 3.01E-01 | 1.14E+00 | 1.75E+00 | 5.77E-01 | 7.82E-02 | 1.02E+01 | 3.24E-01 | 1.33E+00 | 2.05E-01 | 6.59E-03 | 2.03E-01 | 1.98E-01 | 1.03E+00 | 2.21E-01 | 1.61E-01 | 1.61E-02 | 2.39E-01 | 6.59E-03 |
| Orotic acid | 6.57E-01 | 4.30E-01 | 2.86E-01 | 4.45E+00 | 4.72E-01 | 4.65E+00 | 2.14E+00 | 1.28E+00 | 4.44E-01 | 2.94E-01 | 2.90E-01 | 2.86E-01 | 2.86E-01 | 2.86E-01 | 4.95E-01 | 2.86E-01 | 4.55E-01 | 5.07E-01 |
| Oxidized glutathione | 1.36E-03 | 1.14E+00 | 1.61E+00 | 2.64E-03 | 1.36E-03 | 4.90E-02 | 3.80E+00 | 2.99E+00 | 1.36E-03 | 1.32E+00 | 3.03E-01 | 1.02E-01 | 1.49E+00 | 1.66E-01 | 1.06E+00 | 4.01E-01 | 9.49E-01 | 2.59E+00 |
| Pantothenol | 1.97E-01 | 6.02E-01 | 1.08E+00 | 1.45E+00 | 8.97E-01 | 2.09E+00 | 1.14E+00 | 2.10E+00 | 2.64E-01 | 8.18E-01 | 1.74E-01 | 2.59E-01 | 4.16E+00 | 1.74E-01 | 7.62E-01 | 3.14E-01 | 6.23E-01 | 8.99E-01 |
| Petroselinic acid | 6.40E-01 | 6.40E-01 | 6.40E-01 | 6.40E-01 | 6.40E-01 | 6.40E-01 | 1.41E+00 | 6.40E-01 | 6.40E-01 | 8.97E-01 | 7.05E-01 | 8.42E-01 | 1.43E+00 | 1.70E+00 | 2.91E+00 | 6.59E-01 | 6.40E-01 | 1.68E+00 |
| Pipecolic acid | 7.10E-01 | 1.10E+00 | 3.00E+00 | 8.29E-01 | 6.65E-01 | 1.09E+00 | 2.85E+00 | 1.17E+00 | 2.83E-01 | 1.02E+00 | 8.51E-01 | 4.13E-01 | 7.67E-01 | 2.34E-01 | 8.84E-01 | 5.59E-01 | 5.14E-01 | 1.04E+00 |
| Progesterone | 2.91E-01 | 7.04E-01 | 4.26E-01 | 9.41E-01 | 1.35E-01 | 4.25E+00 | 2.39E+00 | 4.44E-01 | 9.91E-02 | 3.36E-01 | 2.96E-01 | 4.48E-01 | 7.75E-01 | 3.59E-01 | 2.64E+00 | 2.26E-01 | 3.99E-01 | 2.84E+00 |
| Prostaglandin E2 | 4.58E-02 | 9.37E-02 | 1.15E-01 | 7.07E+00 | 2.25E+00 | 6.20E+00 | 1.20E+00 | 1.10E-01 | 4.58E-01 | 4.58E-02 | 4.58E-02 | 4.58E-02 | 4.58E-02 | 4.58E-02 | 4.58E-02 | 4.58E-02 | 9.25E-02 | 4.58E-02 |
| Purine | 6.34E-01 | 2.09E-01 | 1.00E+00 | 6.50E+00 | 1.79E-01 | 3.69E+00 | 3.18E+00 | 1.51E+00 | 2.84E-01 | 1.58E-01 | 8.07E-02 | 8.07E-02 | 8.07E-02 | 8.07E-02 | 8.07E-02 | 8.07E-02 | 8.07E-02 | 8.07E-02 |
| Pyridoxal | 5.23E-01 | 7.70E-01 | 8.06E-01 | 3.60E+00 | 4.53E-01 | 2.20E+00 | 3.07E+00 | 3.01E-01 | 1.41E-01 | 3.25E-01 | 4.13E-01 | 1.41E-01 | 8.57E-01 | 1.76E-01 | 8.68E-01 | 4.46E-01 | 1.03E+00 | 1.87E+00 |
| Pyridoxal 5'-phosphate | 1.17E+00 | 8.60E-01 | 1.38E+00 | 3.38E+00 | 7.14E-01 | 3.85E+00 | 2.80E+00 | 3.17E-01 | 3.09E-01 | 2.70E-01 | 3.08E-01 | 1.58E-01 | 3.43E-01 | 1.58E-01 | 4.77E-01 | 1.58E-01 | 5.55E-01 | 7.92E-01 |
| Pyroglutamic acid | 1.21E+00 | 8.47E-02 | 8.47E-02 | 4.19E+00 | 5.90E-01 | 4.55E+00 | 3.94E+00 | 8.47E-02 | 4.80E-01 | 5.34E-01 | 3.28E-01 | 8.47E-02 | 6.72E-01 | 8.47E-02 | 8.47E-02 | 8.47E-02 | 3.09E-01 | 5.90E-01 |
| Retinal | 7.37E-01 | 7.37E-01 | 2.34E+00 | 1.05E+00 | 9.93E-01 | 7.37E-01 | 7.37E-01 | 7.37E-01 | 1.22E+00 | 7.37E-01 | 2.29E+00 | 1.01E+00 | 7.37E-01 | 9.31E-01 | 7.95E-01 | 7.37E-01 | 7.37E-01 | 7.37E-01 |
| Rhamnose | 2.72E-01 | 7.18E-02 | 4.86E-02 | 3.92E-01 | 5.46E-01 | 6.56E-01 | 1.54E-01 | 6.21E-02 | 4.76E-01 | 2.93E+00 | 1.76E+00 | 3.49E-01 | 2.26E+00 | 3.58E-01 | 2.00E+00 | 9.30E-01 | 1.33E+00 | 3.41E+00 |
| Riboflavin | 6.82E-01 | 4.32E-01 | 6.02E-01 | 3.11E+00 | 6.62E-01 | 3.16E+00 | 2.14E+00 | 1.03E+00 | 4.96E-01 | 7.27E-01 | 5.05E-01 | 8.55E-02 | 1.09E+00 | 8.73E-02 | 4.47E-01 | 1.75E-01 | 4.72E-01 | 2.10E+00 |
| Ribothymidine | 7.68E-01 | 4.34E-01 | 8.02E-01 | 2.82E+00 | 1.56E+00 | 4.47E+00 | 3.65E+00 | 1.93E+00 | 8.94E-01 | 5.78E-02 | 1.27E-01 | 5.78E-02 | 5.78E-02 | 5.78E-02 | 1.33E-01 | 5.78E-02 | 5.78E-02 | 5.78E-02 |
| Salicylic acid | 1.22E+00 | 5.19E-01 | 6.99E-01 | 4.07E-01 | 2.14E+00 | 4.07E-01 | 4.07E-01 | 5.50E-01 | 9.78E-01 | 4.07E-01 | 1.21E+00 | 1.39E+00 | 1.28E+00 | 2.80E+00 | 7.18E-01 | 1.77E+00 | 4.07E-01 | 7.09E-01 |
| Sebacic acid | 1.09E+00 | 5.73E-01 | 6.81E-01 | 1.86E+00 | 3.78E-01 | 2.28E+00 | 1.15E+00 | 9.03E-01 | 3.78E-01 | 1.33E+00 | 8.65E-01 | 3.78E-01 | 1.25E+00 | 4.49E-01 | 9.30E-01 | 6.05E-01 | 7.72E-01 | 2.12E+00 |
| Serotonin | 1.92E-03 | 6.12E-03 | 2.51E-02 | 1.64E-02 | 1.62E-03 | 1.62E-03 | 2.38E-02 | 1.80E-02 | 1.62E-03 | 3.19E+00 | 1.34E+00 | 4.28E-01 | 2.64E+00 | 7.85E-01 | 1.61E+00 | 5.71E-01 | 2.05E+00 | 5.28E+00 |
| Sorbitol | 1.44E+00 | 1.32E+00 | 7.06E-01 | 2.36E+00 | 1.22E+00 | 2.54E+00 | 1.22E+00 | 1.05E+00 | 1.04E+00 | 9.74E-01 | 2.08E-01 | 3.93E-02 | 8.72E-01 | 5.79E-02 | 9.75E-02 | 1.20E-01 | 6.84E-01 | 2.06E+00 |
| Sphingosine | 3.95E-01 | 6.68E-01 | 2.85E-01 | 8.33E-01 | 3.07E-01 | 1.37E+01 | 8.23E-01 | 2.76E-01 | 4.42E-01 | 5.64E-04 | 1.47E-02 | 2.12E-02 | 3.52E-02 | 3.13E-02 | 5.98E-02 | 3.40E-03 | 1.17E-02 | 8.35E-02 |
| Succinic acid | 1.08E-01 | 4.67E-01 | 2.27E-01 | 6.50E-02 | 1.40E-01 | 6.50E-02 | 9.79E-01 | 3.27E+00 | 6.50E-02 | 2.40E+00 | 1.04E+00 | 6.50E-02 | 1.82E+00 | 6.50E-02 | 2.76E+00 | 9.22E-01 | 1.14E+00 | 2.41E+00 |
| Taurine | 1.82E+00 | 2.75E+00 | 2.98E+00 | 1.35E+00 | 9.92E-01 | 1.53E+00 | 8.44E-01 | 2.39E+00 | 3.95E-01 | 1.66E-01 | 2.53E-01 | 3.65E-01 | 2.17E-01 | 3.82E-01 | 3.93E-01 | 4.04E-01 | 5.97E-01 | 1.76E-01 |
| Taurocholic Acid | 1.34E-01 | 6.52E-02 | 7.21E-03 | 7.32E-01 | 8.17E-02 | 3.56E-01 | 1.17E-01 | 7.21E-03 | 5.45E-02 | 2.46E-01 | 2.90E+00 | 3.96E-01 | 9.32E+00 | 7.82E-02 | 2.70E-01 | 4.74E-01 | 1.12E-01 | 2.65E+00 |
| Tetradecanedioic acid | 2.18E-01 | 1.41E+00 | 1.08E+00 | 1.62E+00 | 5.63E-01 | 4.71E+00 | 2.18E-01 | 2.30E+00 | 2.46E+00 | 2.18E-01 | 2.18E-01 | 1.67E+00 | 2.18E-01 | 2.18E-01 | 2.18E-01 | 2.18E-01 | 2.18E-01 | 2.18E-01 |
| Theophylline | 9.20E-01 | 3.06E-01 | 4.82E-01 | 1.74E+00 | 4.53E-01 | 8.97E-01 | 9.68E-01 | 7.34E-01 | 6.77E-01 | 3.06E+00 | 9.72E-01 | 1.94E-01 | 2.11E+00 | 5.32E-02 | 1.79E-01 | 1.09E-01 | 8.82E-01 | 3.26E+00 |
| Thymine | 1.12E+00 | 6.84E-01 | 1.18E+00 | 2.94E+00 | 1.54E+00 | 3.57E+00 | 3.08E+00 | 1.76E+00 | 9.20E-01 | 2.15E-01 | 1.55E-01 | 7.02E-02 | 2.61E-01 | 7.02E-02 | 1.10E-01 | 7.02E-02 | 7.02E-02 | 1.94E-01 |
| Thyroxine | 1.30E-01 | 1.53E-01 | 1.30E-01 | 1.30E-01 | 1.30E-01 | 1.68E+00 | 1.30E-01 | 1.30E-01 | 1.30E-01 | 1.20E+00 | 1.55E-01 | 1.30E-01 | 2.21E+00 | 1.55E+00 | 1.94E+00 | 1.50E-01 | 1.24E+00 | 6.69E+00 |
| Tryptamine | 1.26E+00 | 9.45E-01 | 7.01E-01 | 1.34E+00 | 7.01E-01 | 7.81E-01 | 7.01E-01 | 7.32E-01 | 7.01E-01 | 7.01E-01 | 1.01E+00 | 8.35E-01 | 1.51E+00 | 1.60E+00 | 7.01E-01 | 8.67E-01 | 8.16E-01 | 2.10E+00 |
| Tryptophanol | 7.42E-01 | 4.10E-01 | 4.10E-01 | 1.27E+00 | 4.10E-01 | 1.57E+00 | 1.45E+00 | 6.13E-01 | 7.69E-01 | 1.97E+00 | 4.92E-01 | 4.10E-01 | 1.45E+00 | 4.10E-01 | 5.98E-01 | 4.10E-01 | 8.72E-01 | 3.75E+00 |
| Uracil | 2.87E+00 | 1.05E+00 | 1.67E+00 | 5.93E-02 | 1.65E+00 | 5.44E+00 | 5.93E-02 | 2.10E+00 | 1.33E+00 | 3.38E-01 | 1.62E-01 | 7.11E-02 | 4.44E-01 | 5.93E-02 | 2.30E-01 | 1.05E-01 | 1.28E-01 | 2.31E-01 |
| Urea | 1.64E-01 | 9.63E-02 | 9.05E-02 | 3.68E-01 | 1.40E-01 | 1.75E-01 | 1.42E-01 | 2.08E-01 | 2.69E-01 | 2.11E+00 | 1.47E+00 | 3.23E-01 | 2.91E+00 | 2.45E-01 | 9.96E-01 | 6.42E-01 | 1.62E+00 | 6.03E+00 |
| Ureidopropionic acid | 2.48E-01 | 4.75E-01 | 7.35E-01 | 7.62E-01 | 2.17E-01 | 4.12E-01 | 4.81E-01 | 5.84E-01 | 3.72E-01 | 1.71E+00 | 1.25E+00 | 2.33E-01 | 3.42E+00 | 2.24E-01 | 1.08E+00 | 7.04E-01 | 1.36E+00 | 3.73E+00 |
| Uridine | 2.16E+00 | 4.23E-01 | 1.91E+00 | 1.90E+00 | 2.68E+00 | 2.24E+00 | 2.95E+00 | 9.77E-01 | 5.76E-01 | 3.47E-01 | 2.31E-01 | 6.60E-02 | 3.71E-01 | 7.12E-02 | 3.01E-01 | 1.32E-01 | 2.10E-01 | 4.66E-01 |
| Valeric acid | 1.13E+00 | 8.41E-01 | 8.41E-01 | 1.37E+00 | 1.18E+00 | 8.41E-01 | 8.41E-01 | 9.98E-01 | 8.41E-01 | 8.76E-01 | 8.41E-01 | 1.00E+00 | 8.41E-01 | 1.18E+00 | 1.22E+00 | 1.15E+00 | 1.02E+00 | 9.84E-01 |
| Xanthosine | 1.21E+00 | 5.66E-02 | 8.36E-01 | 6.36E+00 | 1.12E+00 | 5.66E-02 | 4.24E+00 | 1.85E+00 | 5.01E-01 | 7.81E-01 | 2.80E-01 | 5.66E-02 | 3.17E-01 | 5.66E-02 | 1.13E-01 | 5.77E-02 | 5.66E-02 | 5.66E-02 |
| Xanthurenic acid | 1.21E-01 | 3.48E-02 | 9.25E-03 | 3.80E-01 | 5.36E-02 | 1.96E-01 | 1.45E-01 | 2.79E-01 | 2.07E-01 | 1.33E+00 | 9.67E-01 | 9.01E-02 | 3.33E+00 | 6.82E-02 | 9.43E-01 | 5.11E-01 | 2.87E+00 | 6.46E+00 |

References

1. Kanehisa, M. & Goto, S. KEGG: Kyoto Encyclopedia of Genes and Genomes. *Nucleic Acids Res.* **28,** 27-30 (2000).
